# Detection of Alpha-Rod Protein Repeats Using a Neural Network and Application to Huntingtin

**DOI:** 10.1371/journal.pcbi.1000304

**Published:** 2009-03-13

**Authors:** Gareth A. Palidwor, Sergey Shcherbinin, Matthew R. Huska, Tamas Rasko, Ulrich Stelzl, Anup Arumughan, Raphaele Foulle, Pablo Porras, Luis Sanchez-Pulido, Erich E. Wanker, Miguel A. Andrade-Navarro

**Affiliations:** 1 Ottawa Health Research Institute, Ottawa, Ontario, Canada; 2 Medical Imaging Research Group, The University of British Columbia, Vancouver General Hospital, Vancouver, British Columbia, Canada; 3 Max-Delbrück Center for Molecular Medicine, Berlin, Germany; 4 Otto-Warburg Laboratory, Max Planck Institute for Molecular Genetics, Berlin, Germany; 5 Functional Genetics Unit, Department of Physiology, Anatomy and Genetics, University of Oxford, Oxford, United Kingdom; University of California San Diego, United States of America

## Abstract

A growing number of solved protein structures display an elongated structural domain, denoted here as alpha-rod, composed of stacked pairs of anti-parallel alpha-helices. Alpha-rods are flexible and expose a large surface, which makes them suitable for protein interaction. Although most likely originating by tandem duplication of a two-helix unit, their detection using sequence similarity between repeats is poor. Here, we show that alpha-rod repeats can be detected using a neural network. The network detects more repeats than are identified by domain databases using multiple profiles, with a low level of false positives (<10%). We identify alpha-rod repeats in approximately 0.4% of proteins in eukaryotic genomes. We then investigate the results for all human proteins, identifying alpha-rod repeats for the first time in six protein families, including proteins STAG1-3, SERAC1, and PSMD1-2 & 5. We also characterize a short version of these repeats in eight protein families of Archaeal, Bacterial, and Fungal species. Finally, we demonstrate the utility of these predictions in directing experimental work to demarcate three alpha-rods in huntingtin, a protein mutated in Huntington's disease. Using yeast two hybrid analysis and an immunoprecipitation technique, we show that the huntingtin fragments containing alpha-rods associate with each other. This is the first definition of domains in huntingtin and the first validation of predicted interactions between fragments of huntingtin, which sets up directions toward functional characterization of this protein. An implementation of the repeat detection algorithm is available as a Web server with a simple graphical output: http://www.ogic.ca/projects/ard. This can be further visualized using BiasViz, a graphic tool for representation of multiple sequence alignments.

## Author Summary

Many proteins have an elongated structural domain formed by a stack of alpha helices (alpha-rod), often found to interact with other proteins. The identification of an alpha-rod in a protein can therefore tell something about both the function and the structure of that protein. Though alpha-rods can be readily identified from the structure of proteins, for the vast majority of known proteins this is unavailable, and we have to use their amino acid sequence. Because alpha-rods have highly variable sequences, commonly used methods of domain identification by sequence similarity have difficulty detecting them. However, alpha-rods do have specific patterns of amino acid properties along their sequences, so we used a computational method based on a neural network to learn these patterns. We illustrate how this method finds novel instances of the domain in proteins from a wide range of organisms. We performed detailed analysis of huntingtin, the protein mutated in Huntington's chorea, a neurodegenerative disease. The function of huntingtin remains a mystery partially due to the lack of knowledge about its structure. Therefore, we defined three alpha-rods in this protein and experimentally verified how they interact with each other, a novel result that opens new avenues for huntingtin research.

## Introduction

**Figure 1:**
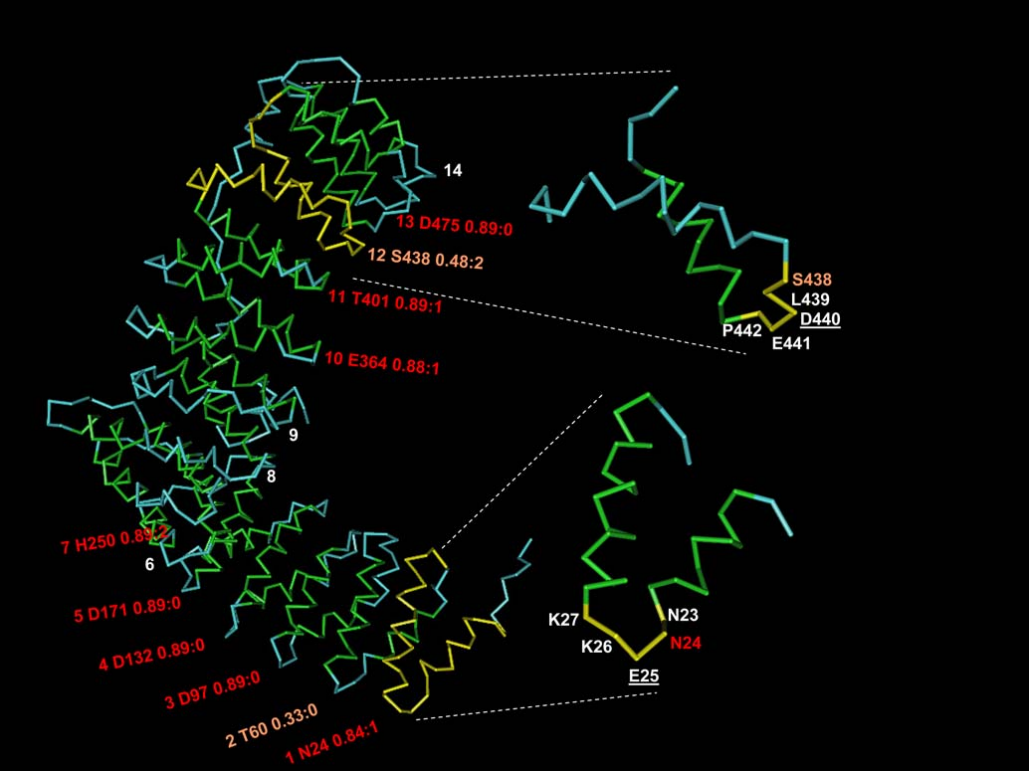
Detection of repeats in an alpha-rod protein. Structure (alpha-backbone trace) of the 591 aa N-terminal fragment of human adaptor-related protein complex 2, beta 1 subunit, as forming part of the AP2 clathrin adaptor core [69] (PDB code 2VGL chain B). Green and blue represent residues in alpha-helix and in disordered conformation, respectively. This structure has no residue in beta-strand conformation and is entirely composed of an alpha-rod of 14 repeats previously classified as HEAT repeats of type ADB [4]. The label for each repeat indicates the following: repeat order, residue detected by the network, score of hit, and position relative to residue used for training. For example, “1 N24 0.84∶1” indicates that the residue detected for repeat #1 was N (amino acid code for asparagine) in position 24 of the sequence, with score 0.84, but that the residue in relative position 1 (that is, at 25) was the one used to train the network as being in the hinge. Ten out of the 14 repeats were detected, 8 of them with score> = 0.80. The inset shows repeats 12 (right, top) and 1 (right, bottom) with the residue used as positive in the training underscored. A coloured label indicates the residue identified by the network after training, which in both cases is not the one given in the training but others belonging to the hinge (E25 and S438). The figure was generated using NCBI's linked viewer, Cn3D [70].

Tandems of repeated protein sequences forming structural domains occur in at least 3% of proteins in eukaryotic organisms [1]. Characterization of these repeats by sequence similarity is sometimes difficult as weak evolutionary constraints cause rapid sequence divergence [2]. In particular, repeats including two alpha helices packed together then stacked to form a flexible rod (denoted here alpha-rod) belong to this category (see an example in Figure 1).

Some of these alpha-rod repeats have been defined in terms of sequence similarity and are widespread in multiple protein families: HEAT [3],[4], Armadillo [5] and HAT [6]. Others are evident in just one protein family, for example the PFTA repeats [7]. Some, however, bear no statistically significant sequence similarity and may not have originated from sequence duplication (for example, the all-helical VHS domain in *Drosophila melanogaster* Hrs protein [8], or the subunit H of *Saccharomyces cerevisiae* vacuolar ATP synthase [9]).

This divergence complicates the detection of alpha-rod repeats by methods based on sequence similarity. For example, profile-based methods used in the protein domain databases PFAM [10] and SMART [11] detect only two of the 14 HEAT repeats of human AP-2 complex subunit beta-1 (Figure 1), and might fail to detect any repeats in other alpha-rod containing sequences.

Despite the heterogeneity of alpha-rod repeats, they have common features (discussed in [4]): length of about 40 amino acids, anti-parallel alpha-helices, and constraints given by the packing of consecutive repeats. This suggests that alpha-rod repeats are a protein structural feature that obeys some physical constraints irrespective of their evolutionary origin and particular sequence. Coiled coils and transmembrane alpha-helices are other examples of such structural features. Statistical methods have been used to predict coiled coils [12] and transmembrane alpha-helices [13] with excellent reliability, using algorithms that learn to recognize these features from amino acid sequences. In particular, back-propagation neural networks [14] have been used with success to predict secondary structure [15],[16], transmembrane alpha-helices [17], and protein residue solvent accessibility [18].

We hypothesized that a back-propagation neural network could be better suited than homology based methods for the detection of different types of alpha-rod repeats, if trained in an appropriate set of sequences containing these repeats. The last ten years have seen the resolution of a sufficient number of protein 3D structures of sequences with alpha-rod repeats to provide a useful training set for such predictions.

## Results

We manually compiled a set of protein sequences with known structures reported to contain structural repetitions forming an alpha-rod composed of stacked repeats (see supplementary Table S1 in Text S1, positives). To reduce redundancy, no two sequences with more than 70% identity were included in the set (after verifying that they were full length homologs). We included one protein from each of three HEAT repeat types [4], two armadillo repeat proteins, and five other unrelated proteins. A similar sized set of sequences adopting a variety of structures but without alpha-rod repeats was compiled as a negative set (Table S1 in Text S1, negatives).

The input window of the neural network was chosen to be 39 amino acids, which is close to the average repeat length. Since these repeats are characterized by two helices of similar size, we chose as the central defining feature the middle residue in the hinge between the two helices. This residue should be equidistant from two secondary structure elements with particular packing features, likely presenting a periodicity of small and hydrophobic residues constrained by the intra-repeat interactions between the two helices and the inter-repeat interactions with the stack of consecutive repeats [4]. Therefore, the network was trained to detect the central residue of the hinge (see Methods). The file with the annotated sequences used for the training is provided as supplementary Dataset S1.

### Analysis of Proteins of Known Structure

The parameters of the method were optimized using the analysis of proteins of known structure. We found that hits above a score of 0.8 were reliable, especially when the protein had several of them in the appropriate periodicity. Identification of a sequence as containing an alpha-rod was optimal when requiring at least three hits above a score of 0.8 with a minimum spacing of 30 amino acids between hits and a maximum of 135. Further details can be found in the supplementary Text S1.

A total of 87 sequences were selected with this threshold, which can be grouped in 12 protein families of which 8 were not homologous to those used in the training set (Table S2 in Text S1). Since these examples correspond to proteins of known structure, it was easy to visually verify that of those eight families seven were true positives and only one constituted a false positive. Homology of these proteins to the ones used in the training is extremely low or statistically non-significant. Therefore, we concluded that the network was useful in expanding our current knowledge of the occurrences of these repeats and we set to demonstrate this. For simplicity we will denote our methodology as ARD (Alpha-rod Repeat Detection) henceforth.

### Analysis of Complete Genomes

To illustrate the coverage of the method we analyzed the complete protein sets from a series of fully sequenced organisms. The threshold tested in the analysis of PDB was used to select positive sequences. The results of the analysis are in Table 1. The fractions of alpha-rod repeat proteins are around 0.4% for the nine eukaryotic genomes and lower (0.05%–0.21%) in the three prokaryotic organisms tested. No correlation was found between proteome size and fraction of positives.

**Table 1:** Results of predictions in complete genomes.

Organism	Proteins	Hits^1^	Genes	Fraction
*Homo sapiens*	43797	159	86	0.36%
*Mus musculus*	32241	125	93	0.39%
*Monodelphis domestica*	32685	131	81	0.40%
*Gallus gallus*	22250	102	75	0.46%
*Xenopus tropicalis*	28324	96	69	0.34%
*Danio rerio*	36078	116	85	0.32%
*Drosophila melanogaster*	19789	52	41	0.26%
*Saccharomyces cerevisiae*	6697	23	23	0.34%
*Gibberella zeae*	11640	37	37	0.32%
*Escherichia coli*	4133	2	2	0.05%
*Anabaena variabilis ATCC 29413*	5634	11	11	0.20%
*Methanosarcina mazei*	3303	7	7	0.21%

1 At least three matches with score> = 0.8 and with > = 30 aa spacing.

Using ARD we were able to detect protein sequences that PFAM [10] and SMART [11] do not detect or that they detect with multiple profiles (PFAM: Arm, HEAT_PBS and HEAT; SMART: ARM, EZ_HEAT and HEAT). Many of these were not described in the literature.

To illustrate the ability of ARD to identify new results we will focus on families with at least one human gene. To illustrate how the method covers various profiles used by SMART and PFAM we will examine results on families with HEAT repeats of the PBS type from fungi, bacteria, and archaea. Finally, we illustrate an experimental application of the method to dissect domains in huntingtin, the protein mutated in Huntington's disease, for which little is known regarding its structure and function.

### Survey of Human Genes

A total of 86 human proteins were found to contain alpha-rod repeats, which we grouped in 52 families on the basis of their sequence similarity. Of those families, at least 16 have not been yet described to contain alpha-rod repeats in the literature, with 9 undetected by both the SMART and PFAM domain detection web tools (see Table 2).

**Table 2:** Selected predictions for human genes^a^.

	Representative	Description	A^b^	S	P	R	Ref^c^	Related^d^
Novel	STAG1	Homologs of *yeast* subunit of the cohesin complex 3 (Scc3/IRR1)	4	0	0	0	u	STAG2 STAG3
	SERAC1	serine active site containing 1	3	0	0	0	u	
	C8orf73		5	0	0	0	u	
	C17orf66		6	0	0	0	u	
	KIAA0423	LOC23116	11	0	0	5	u	
	PSMD1	proteasome 26S subunit, non-ATPase family	5	0	0	0	u	PSMD2 PSMD5
No PFAM/SMART	MRO	Maestro	3	0	0	0	[30]	NP_775760.2 Q8NDA8_HUMAN Q8ND95_HUMAN
	NIPBL	Nipped-B homolog (Drosophila)	7	0	0	5	[31]	
	FRAP1	FRAP1/mTOR	16	0	0	11	[3]	
Domain redefinition	RRP12	Ribosomal RNA processing 12 homolog (yeast)	9	1	1	0	[28]	
	CLASP1	CLASP family	10	4	4	7	[38]	CLASP2
	CKAP5	CKAP5	18	3	3	10	[31]	
Not reported in literature	KIAA1468		6	0	3	0	u	
	HEATR2		9	6	6	11	u	
	HEATR4		9	0	3	0	u	
	HEATR6		7	2	2	4	u	
	TMCO7		7	0	2	0	u	
	STK36	Serine/threonine kinase 36, fused homolog (Drosophila)	7	3	3	0	u	
	INTS4	integrator complex subunit 4	8	5	5	5	u	Q96LV5_HUMAN
	RTDR1	Rhabdoid tumor deletion region protein 1	6	0	1*	0	u	
	LOC165186		7	1	1	0	u	
	C1orf175		6	0	1	0	u	

a Hits not included: reported in the literature (SF3B1, MMS19, huntingtin, PSME4, NCAPD3, NCAPG2, TBCD, BTAF1, KOG1, PDS5B); armadillo repeats (JUP, RAP1GDS1); likely false positives (OBSCN, P2RY8, PACS2); other genes homologous to 3D structures discussed before.

b Predicted number of repeats by ARD (A), SMART (S), PFAM (P), REP (R).

c Reference column. “u” indicates unknown from the point of view of the literature.

d Close homologous genes are indicated.

*: Armadillo repeat.

**Figure 2:**
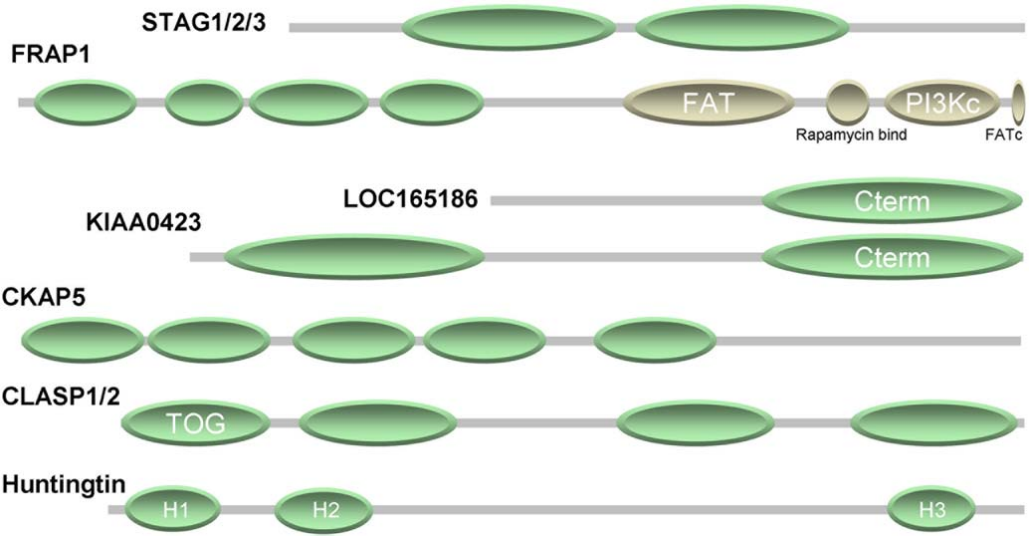
Selected human protein families with alpha-rod repeats. The cartoon summarizes the findings for seven human proteins. The green ellipses represent regions of alpha-rod repeats as deduced by a combination of our method, analysis of homologs, and iterative sequence analysis. Further details for each case, including an overview of repeat predictions and regions with amino acid bias overlaid to the multiple sequence alignment of the family using an update of the BiasViz software [71] are available as supplementary Figure S3 in Text S1.

In particular, six families have neither literature nor database repeat assignment; for these, we could verify the repeats using a manually tuned iterative PSIBLAST sequence search [19] of the region with repeats, which showed significant similarity to alpha-rod repeat regions in other protein families. Four of these families encode proteins of unknown function: Serac1, C8orf73, C17orf66, and KIAA0423 (and homolog LOC23116). A fifth family has three members in humans, the stromal antigens 1, 2 and 3 (STAG1-3), subunits of the cohesin complex, which mediates cohesion between sister chromatids [20]. In particular, the phosphorylation of STAG2 is essential for cohesin dissociation during prophase and prometaphase [21]. This family has two homologs in *Xenopus* (demonstrated to form part of two different cohesion complexes [22]), the plant *Arabidopsis thaliana* (Scc3, needed for the orientation of the kinetochores during meiosis [23]) and yeast (Irr1/Scc3, involved in cell wall integrity [24]). The analysis of the family suggests that their sequences are composed of alpha-rod repeats (Figure 2 and Figure S3A in Text S1).

The sixth novel assignment case is the PSMD family (proteasome 26S subunit, non-ATPase) members 1, and 2, and 5. PFAM/SMART identify these as containing repeats of the Proteasome/cyclosome (PC_rep), originally predicted to be composed of a beta strand and a alpha helix [25]. However, ARD predicts 5 repeats which overlap with those. Secondary structure predictions (using JPRED3 [26]) and homology to alpha-rod repeats proposed for PSMD1 yeast homolog Sen3/RPN2 [27] clearly suggest that these are alpha-rod repeats, and that the current PC_rep motif used by PFAM/SMART cuts one of the helices in half. This suggests that the PFAM/SMART domain definition should be revised.

Another family for which a redefinition of the PFAM/SMART profile may be required is RRP12, homolog to the yeast Ribosomal RNA processing 12, identified as HEAT-repeat containing, Ran binding, and required for the nuclear export of both the 40S and 60S ribosomal subunits in yeast [28]. SMART and PFAM identify only one HEAT repeat in the human sequence because other repeats overlap with domain NUC173, defined as present in several nucleolar proteins [29], whereas ARD identifies 9 repeats.

Three other families remain undetected by PFAM and SMART profiles but have been described to contain alpha-rod repeats in separate publications: these are the MRO (Maestro), which expresses a nucleolar protein of unknown function during male mouse gonad development [30], FRAP1/mTOR, which we described as repeat containing in the first publication defining the HEAT repeats [3] (Figure 2 and Figure S3B in Text S1), and NIPBL (the homolog to *Drosophila* Nipped-B) related to sister chromatid cohesion yeast proteins Scc2 and Mist4 [31].

For ten other gene families, PFAM and SMART suggest the presence of the repeats but their coverage is more limited than that of ARD and this evidence remains unreported in the literature. This is the case of STK36/FU (the homolog to *Drosophila* fused, a mediator of sensitivity to PARP [32]), INTS4 (integrator complex subunit 4, which associates with the C-terminal domain of RNA polymerase II large subunit [33]), and of eight hypothetical proteins: C1orf175, LOC165186, HEATR2, HEATR4, HEATR6, KIAA1468, RTDR1 (deleted in rhabdoid tumour), and TMCO7 (which interacts with MACF1, the microtubule-actin crosslinking factor 1 according to a two-hybrid screening [34]).

The combination of ARD analyses of the human protein homologs in other organisms, secondary structure prediction and definition of regions of amino acid composition bias facilitates the definition of the boundaries of domains composed of repeats sometimes reused in different domain architectures. Here we present three examples.

We found that the LOC165186 and KIAA0423 hypothetical human proteins (mentioned above) define two families whose structured sequence is likely alpha-rods; these two proteins share a C-terminal domain possibly made of more than 10 repeats (Figure 2 and Figure S3C in Text S1). LOC165186, conserved in mammals, has an additional N-terminal composition biased region of around 500 amino acids, whereas KIAA0423, conserved down to worms, has an extra N-terminal domain of alpha-rod repeats connected to the C-terminal repeat domain by a middle linker that is enlarged in the chordate sequences.

Human CKAP5/TOG (cytoskeleton associated protein 5), a component of the centrosome that is required for spindle pole assembly [35], has similar-length homologs in mammals, frog, and fly. Analysis of the family identifies five alpha-rods of six repeats each in these sequences and a C-terminal non-repeat containing domain (Figure 2 and Figure S3D in Text S1). The worm homologs are shorter since they have only three of the repeat domains. The structure of one of those domains in *Caenorhabditis elegans* zyg9 was solved and confirmed the presence of an alpha-rod of six repeats [36].

The CLASP family proteins are microtubule-associated proteins, conserved in animals, fungi, and plants [37]. In humans, there are two homologs, hCLASP1 and hCLASP2, which, similar to CKAP5, associate with the ends of growing microtubules to participate in mitotic spindle formation [38]. Their multiple sequence alignment with homologs suggests that they are formed by four alpha-rods (Figure 2 and Figure S3E in Text S1), also noted in [38].

Other genes previously identified in the literature and by SMART/PFAM are: TBCD (tubulin folding cofactor D) reported by [31]; PSME4/PA200, identified as containing 18 HEAT-like repeats in [39]; BTAF1 (RNA polymerase II, B-TFIID transcription factor-associated, 170 kDa) whose homolog in yeast, Mot1, was noted by [31]; MMS19, involved in nucleotide excision repair and transcription, noted by [40]; huntingtin [3]; both subunits of non-SMC condensin II complex D3 and G2, noted by [31]; and PDS5B/APRIN, a chromatin regulator in hormonal differentiation [41], whose homolog Spo76 in *Sordaria macrospore* was noted by [31].

The existence of two cases where the evidence of repeats originates from low resolution electron microscopy images deserves special mention. SF3B1 (splicing factor 3b, subunit 1) is proposed to have 22 repeats according to the structure obtained by single-particle electron cryomicroscopy at a resolution of less than 10 angstroms of its complex with splicing factor 3a (SF3B14/P14) where it is shown to coil around SF3B14 [42]. The low resolution electron microscopy structure of the yeast complex of mTOR with KOG1 suggests that KOG1 has a middle alpha-rod domain [41]. We can confirm through ARD analysis that both SF3B1 and KOG1 have alpha-rods in the regions suggested.

As noted in the section on analysis of PDB, armadillo repeats are not well detected by ARD and generally PFAM and SMART are as good or better than ARD in recognizing them (for example, for JUP and ARMC8). However, two genes are detected by ARD that are covered by one single PFAM armadillo match and no SMART matches: these are HSPBP1 (hsp70-interacting protein) whose solved 3D structure indicates four armadillo repeats [43] and newly identified RTRD1, for which we detect 3 and 6 repeats, respectively.

Finally, of all 52 protein families with human genes we recognized just three false positives: PACS2 (phosphofurin acidic cluster sorting protein 2), OBSCN (obscurin, cytoskeletal calmodulin and titin-interacting RhoGEF), and P2RY9 (purinergic receptor P2Y, G-protein coupled, 8). This was determined by lack of further evidence (no homology to regions with repeats in other families, incompatible secondary structure predictions) combined with a small number of hits in the human sequence, in homologs in other species, or by the overlap of those hits with other domains.

### Short Repeats Highly Identical within Protein Sequences

In the results of fungal and prokaryotic sequences, we noted a number of cases where the repeats identified for the sequences selected were so similar that it was possible to align most of the repeats by hand in stark contrast to the very divergent examples noted above. We illustrate these with 8 examples, which are not related by homology (see Table S3 in Text S1). Their high percentage of inter-repeat sequence identity is indicative of very recent events of duplication occurring independently in these eight examples. Secondary structure prediction suggests that the structure of the repeat is composed of two helices of ∼10 residues, with a middle loop of three, and an outer loop of ∼10 residues, for a total length of 31–35 aa.

Although most of the repeats were identified by SMART and PFAM (EZ_HEAT and HEAT_PBS profiles, respectively), not all repeat instances were marked and some were detected with the alternative HEAT profile. In contrast, ARD identified all obvious repetitions and some additional borderline ones.

Orthologs of these eight examples were identified in related taxa (Table S3 in Text S1). The puzzling question remains of why or how these eight apparently unrelated families arose and converged to these short alpha-rod repeats. Whether there are common mechanisms for the duplication and selection of these repeats and for their functions is, at the moment, unclear.

### Dissecting Huntingtin

The human protein huntingtin is involved in Huntington's disease. Its function remains unclear [44]. In 1995 we described that huntingtin contains HEAT repeats [3] but their identification was restricted to 10 units covering ∼400 scattered amino acids out of a total sequence length of 3144 amino acids. Since then, no other characteristic structural features have been described for this protein, which complicates its description in terms of separate domains with independent folds and functions. As a result no 3D structure of any fragment of this protein has been yet solved, and although interacting partners of this protein have been found they are mostly restricted to the N-terminal 500 amino acids of the protein [45]. Here, we applied the methodology described above to define alpha-rods in huntingtin and subsequently tested the validity of our predictions experimentally.

Initially, we produced an alignment of human huntingtin with a representative set of homologous sequences from the database (provided as supplementary Dataset S2). For this we used not only sequences from protein databases but also sequences derived from ESTs and from genomic fragments. We identified for the first time the existence of huntingtin homologs in worms (nematoda genus *Caenorhabditis*, and annelida *Capitella sp.*), amoebae (*Naegleria fowleri* and *Dictyostelium discoideum*), sea anemone *Nematostella vectensis*, and choanoflagellate *Monosiga brevicollis*, notably expanding the scope of this family. We did not find homologs of huntingtin in fungi.

The analysis of human huntingtin by ARD suggests six matches but other low scoring hits are consistently present in homologs. Comparison to biased regions sharply defines two N-terminal domains of six and seven repeats (H1 from amino acid 114 to 413 and H2 from 672 to 969) and suggests the existence of a C-terminal domain of seven repeats (H3 from 2667 to 2938) (Figure 2 and Figure S3F in Text S1). Iterative sequence searches using PSIBLAST with these regions indicated homology to HEAT repeats in otherwise unrelated proteins in the 2^nd^ or 3^rd^ iterations. Consistently, sequence analysis suggested a HEAT-repeat fold (using SVMfold [46]), and threading suggested that those regions adopt a HEAT-repeat fold with high likelihood (using GenTHREADER [47]). The comparative protein structure modeling tool TASSER-Lite [48] produced an alpha-rod for H1 and H2, but an alpha-beta barrel for H3 (incompatible with the predicted secondary structure of the region using JPRED3 [26]). Given secondary structure predictions and scattered matches it is tempting to speculate that other alpha-rods exist outside of the H1, H2, and H3 domains. However, we were unable to obtain consistent results using PSIBLAST or threading for fragments outside these regions.

**Figure 3:**
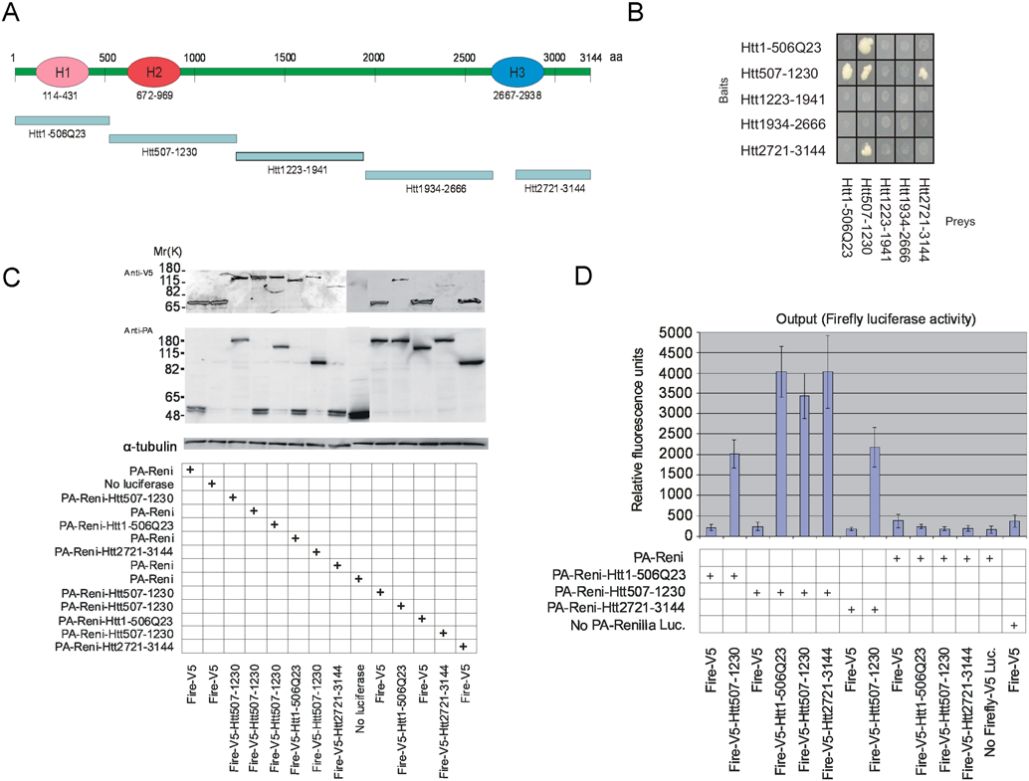
Study of interactions between fragments of huntingtin. (A) Schematic overview of huntingtin fragments used in Y2H and LUMIER experiments. (B) The results obtained with the Y2H assays. (C) The expression of different fusion pairs was analyzed by Western blot using antibodies against V5-epitope (Invitrogen, 1∶5000, monoclonal antibody) and Protein-A (Sigma 1∶2000, polyclonal antibody); 15 µl from 100 µl of each cell extract was loaded onto SDS-PAGE gel. Detection with anti-tubulin antibodies was used as a loading control. (D) Firefly luciferase activities of immunopurified protein complexes in relative fluorescence units (RFU).

To test our predictions, we produced huntingtin fragments spanning the complete sequence of the protein but separating the predicted alpha-rods into different fragments (Figure 3A) in order to study intra-molecular domain interactions in huntingtin by yeast two hybrid (Y2H) assays (see Methods). Our rationale is that only well defined domains will fold and produce interactions, whereas wrongly defined domains will either not interact or produce nonspecific interactions.

We found that the huntingtin fragment Htt507-1230 with the H2 domain self-associates in the Y2H assays. In addition, interactions between Htt507-1230 and Htt1-506Q23 (H1 domain) as well as with the fragment Htt2721-3144 (H3 domain) were observed (Figure 3B). No other interactions were observed.

The results obtained with the Y2H assays were also confirmed in mammalian cells using a modified version of the LUMIER method (luminescence-based mammalian interactome mapping technology, [49]). Protein A (PA)-Renilla luciferase- and Firefly-V5 luciferase (Luc)-tagged huntingtin fusion proteins were co-expressed in HEK293 cells and were assessed for the expression of the fusion proteins by immunoblotting and luciferase assays (Figure 3C and 3D). The PA-Renilla-tagged fusion protein is then immunoprecipitated from the soluble cell extracts with IgG coated Dynal magnetic beads. After washing, binding of the Firefly-V5 Luc-tagged fusion protein is quantified by measuring the firefly luciferase activity in a luminescence plate reader. As shown in Figure 3D, interactions between the huntingtin fragments Htt1-506Q23 and Htt507-1230, Htt507-1230 and Htt507-1230, Htt507-1230 and Htt2721-3144 were observed with the assays.

**Figure 4:**
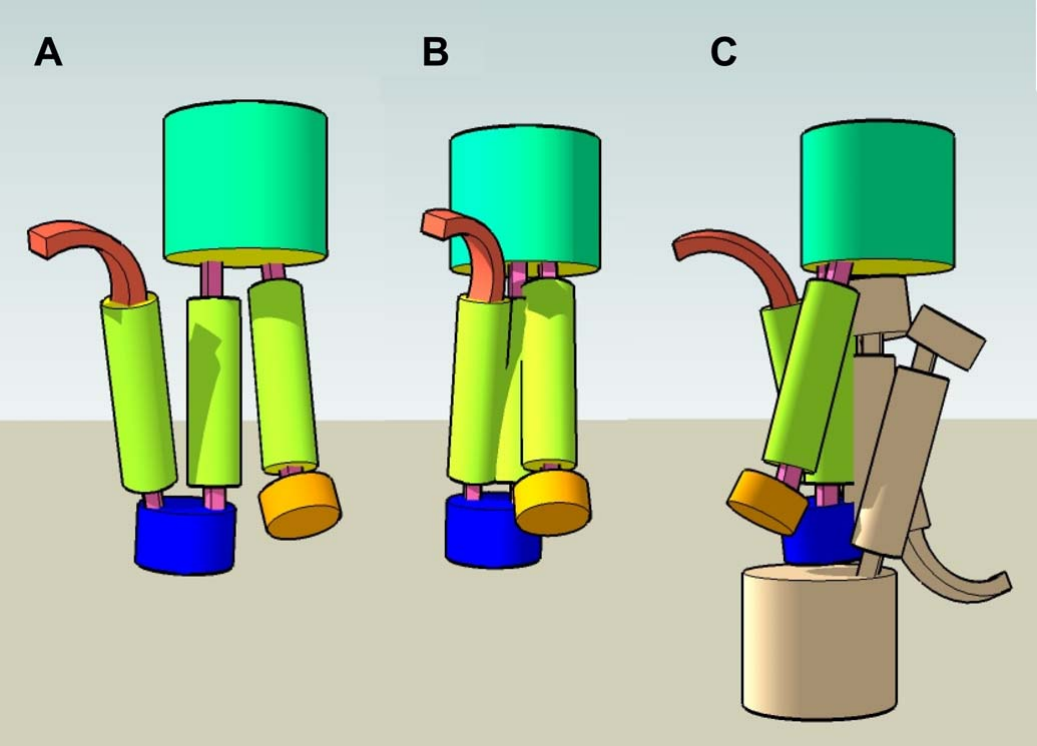
Hypothetical 3D structure of huntingtin. The cartoon represents a hypothetical model of huntingtin interactions consistent with our results. (a) The N-terminus with the poly-Q tail (red arch) is followed by the H1 alpha-rod domain (residues 114 to 431, yellow cylinder), a small domain (432 to 671, blue), the H2 alpha-rod domain (672 to 969, yellow), a large domain (970 to 2666, green), the H3 alpha-rod domain (2667 to 2938), and a small C-terminal domain (2939–3144). (b) The three rods could assemble by coiling anti-parallel to each other with H2 in the middle: that would explain the interactions between H1 and H2, and between H2 and H3. (c) Formation of a huntingtin homodimer [66] with a second molecule of huntingtin (gray) could happen through their H2 domains. The N-terminal poly-Q tail and the H1 domain remain exposed and can interact with other proteins, as previously reported [45]. The figure was produced with Google SketchUp.

Taken together, these experimental results give the first evidence of domains in huntingtin that mediate potential intra- as well as inter-molecular huntingtin interactions. One of many plausible structural assemblies of huntingtin's domains that are consistent with our results and with those in the literature is discussed in Figure 4.

## Discussion

### Performance of the Method

We have developed and applied a neural network for the prediction of alpha-rod repeats. Analysis of the results suggests that it discovers more repeat-containing proteins and repeats per protein than sequence similarity based methods using manually curated profiles, which were previously the best method to detect these repeats. We estimate a level of false positives below 10%: 1 in 12 families in the analysis of PDB (approximately 8%), 3 in 52 families in the analysis of human genes (below 6%). The level of false negatives could be eventually reduced by expanding the training set after new structures of sequences with alpha-rod repeats are solved, but one must be cautious about this to avoid over-prediction. Here, we preferred to train the neural network with a conservative set of known structures to demonstrate that they allow detection of recently identified cases.

We consider it very encouraging that the network learned from a small number of examples and generalized to recognize repeats not used in the training, e.g. the shorter PBS lyase repeats, or those found for the first time in six human protein families. Most of the repeats detected correspond to HEAT, PBS, and Armadillo.

Whereas the network effectively detected a number of unrelated alpha-rod repeat types, it failed to detect the HAT repeats [6]. Although their length is similar, their structural arrangement in highly parallel helices [50] and the conservation of aromatic residues [51] make them significantly different from HEAT and Armadillo repeats explaining why they cannot be detected by our method.

The performance of PFAM, SMART and ARD in predicting each type of alpha-rod repeats in sequences deposited in the PDB database is summarized in Table 3. ARD outperforms PFAM and SMART in the detection of HEAT and PBS repeats but underperforms in the detection of Armadillo repeats (although it identifies some proteins with Armadillo repeats that escape detection by both PFAM and SMART, see Table S2 in Text S1). The proteins in PDB that are currently annotated with HAT repeat regions are detected exclusively by SMART.

**Table 3:** Evaluation of the predictions of PFAM, SMART and ARD, for all proteins in the PDB with four types of alpha-rod repeats.

	PFAM^1^	%	SMART^2^	%	ARD	%	Total
HEAT	21	36	0	0	58	100	58
PBS	1	25	1	25	3	75	4
Armadillo	44	80	50	90	28	50	55
HAT	0	0	7	100	0	0	7

1 PFAM profiles used were PF02984 (HEAT), PF03130 (PBS), PF00514 (Armadillo) and PF02184 (HAT).

2 SMART profiles used were SM00567 (PBS), SM00185 (Armadillo) and SM00386 (HAT).

### Evolutionary and Structural Implications

The lack of a common evolutionary origin for all repeats forming alpha-rods indicates that some specific constraints drive convergent evolution to repeatedly rediscover these repeats as a common solution to a general functional need: protein–protein interactions. Structures of alpha-rods suggest that they are extremely flexible and this allows the ensemble to coil around their target as a boa constrictor would do with its prey. A good example is given by the structure of Exportin Cse1p in complex with Kap60p and RanGTP, where both Cse1p and Kap60p are alpha-rods which wrap around each other, and Cse1p wraps around RanGTP [52].

The necessity to coil around proteins possibly explains why the length of these repeats varies between 30 and 45 amino acids. Shorter repeats might not produce enough interactions between the units to form the rod; consequently the rod would not be stable enough and would unfold too easily. Longer repeats might not produce a rod flexible enough to coil around typical protein targets of diameters in the range of 30 to 50 angstroms.

The current data from protein structures and the predictions of protein domains for proteins with alpha-rods (See Table S2 in Text S1) does not suggest the co-occurrence of alpha-rods with other protein domains. We think that this constitutes further evidence that alpha-rods can be used pretty much to bind any protein as needed.

### Functions of Proteins with Alpha-Rods

Neuwald and Hirano identified in [31] several novel HEAT-repeat containing proteins with functions related to chromosomal organization and microtubule interaction. In agreement with this, here we have identified many alpha-rod repeat containing sequences with related functions, notably direct tubulin binding.

A well characterized example is the TOG domain (an alpha-rod of HEAT repeats), which binds tubulin heterodimers to assist addition of tubulin to the plus-end of microtubules [53]; the crystal structure of the TOG domain in *Caenorhabditis elegans* Zyg9 suggests how this interaction may happen through intra-repeat turns [36]. There is evidence of other microtubule-interacting sequences with alpha-rod repeats: yeast Stu2p binds tubulin [36], clathrin-coated vesicles are assembled along microtubules [54], the protein phosphatase 2A (PP2A) binds to microtubules [55], armadillo-repeat containing sperm antigen 6 (Spag6) colocalizes with microtubules [56] (its homolog in *Chlamydomonas reinhardtii* is PF16, involved in protein–protein interactions required for microtubule stability and flagellar motility [57]), huntingtin association with microtubules was initially found in vitro [58] and then with the beta subunit of tubulin in vivo [59].

A particular case is the plant specific family Tortifolia1/TOR1/SPR2, first characterized in *Arabidopsis thaliana* as microtubule-associated protein and containing HEAT repeats [60]. Its N-terminal HEAT repeat domain has been proven to bind to tubulin [61]. Our analysis suggests that this domain possibly contains seven repeats and is distantly related to the CLASP family (data not shown). Several non-plant protozoan sequences (in amoeba *Dictyostelium discoideum*, and in ciliates *Paramecium tetraurelia strain d4-2* and *Tetrahymena thermophila SB210*) are more similar to the plant family than to distantly related metazoan members hinting at a complex evolution for this family, possibly involving horizontal transfer events between plants and protozoa (data not shown).

Other proteins with alpha-rod repeats not known to be directly involved in interaction with microtubules or tubulin have broadly associated functions: excess importin-beta blocks kinetochore-associated microtubule formation and enhances centrosome-associated microtubule formation [62], STAG/Scc3 localizes to the spindle poles during mitosis and interacts with NuMA, a spindle pole-associated factor required for mitotic spindle organization [60].

This evidence further confirms a general function of eukaryotic alpha-rods in the organization of cellular structure, chromosome segregation, vesicular transport, and control of cell division by protein–protein interactions that tend to involve the microtubules if not tubulin subunits directly.

### Study of Huntingtin

We demonstrated how to combine information from homologous proteins and secondary structure predictions for a better definition of domains of repeats. We used this approach to define three domains of alpha-rod repeats in human huntingtin: H1 between positions 114–413, H2 between 672–969, and H3 between 2667–2938 (Figure 3A). The definition of these three domains correlates well with previous definitions of cleavage sites in huntingtin. In striatum of brains from patients of Huntington's disease a 40–50 kDa N-terminal and a C-terminal 30–50 kDa fragment are observed [63], which would include H1 and H3, respectively. In addition, several caspase cleavage sites have been verified for huntingtin in positions 513, 552 and 586 [64], which fall in between predicted H1 and H2 alpha-rods.

Using our predictions, we verified for the first time interactions between domains of human huntingtin. These involve three domains of HEAT-repeats. Interactions between domains composed of HEAT-repeats are known. For example, several of the subunits of the AP1 clathrin adaptor core are an alpha-rod of HEAT-repeats and interact with each other [65]. We observed the self-association of one of the huntingtin fragments containing a HEAT-repeat domain. This suggests the possibility that huntingtin homodimerizes through inter-molecular association of this domain, in agreement with previous reports [66]. Homodimerization through interaction of domains with HEAT repeats has been suggested for the DNA-PKc/Ku70/Ku80 complex [67].

The interaction of these domains implies their folding in functional units that correspond to the boundaries we have defined. These results are the first demonstration of domains in huntingtin. This opens avenues for further research into the structure and function of this large protein, which had been hampered until now by its lack of definition in terms of structural units. It is now possible to study the interaction of huntingtin with other proteins on a per domain basis.

### Conclusion

We have provided a way forward for the description of these elusive repeats that will facilitate the characterization of domains, structures, and eventually functions of a large number of proteins, possibly up to 0.5% of the proteomes of eukaryotic organisms. Further work is needed to expand the scope of the method, for example to detect HAT repeats and conceivably other as-yet undiscovered alpha-rod repeats. To facilitate the use of the method we have made it available at http://www.ogic.ca/projects/ard. Results of the analysis of protein families can be studied together using ARD in combination with secondary structure predictions via an updated version of our BiasViz multiple sequence alignment viewer (http://biasviz.sourceforge.net).

## Methods

### Neural Network

We used a neural network of feed-forward type with three layers of neurons [14]. Inputs were obtained by scanning the sequence with a 39 amino acid window. The encoding procedure converts the sequence into a binary string where each amino acid is codified by the binary pattern. The length of the entry layer is 39 times 20, where 20 is the number of possible amino acids. One hidden layer with three neurons is used for connecting the inputs with the output layer containing one neuron predicting whether the window is on a repeat or not (e.g. takes real values from 0.1 to 0.9 where the larger values indicates the larger probability of the repeat detection). This architecture was found to be optimal in terms of recall and precision on the training set and computation time required for training and evaluation. Further details of algorithm and training procedure are available in the supplementary Text S1.

### Cloning of Huntingtin Fragments

DNA fragments coding for huntingtin fragments separating predicted domains of alpha-rod repeats were generated by PCR amplification using pAC1-HD plasmid as template. PCR reactions contained, in a 50 µl volume, ∼50 ng plasmid DNA, 15 pmol primer oligonucleotides, 20 mM TRIS-HCl pH 8.8, 2.5 mM MgCl_2_, 50 mM KCl, 10 mM 2-mercaptoethanol and 2.5 U Pwo DNA polymerase (Sigma). Fragments were amplified in 30 cycles with the following profile: 60 s denaturation at 94°C followed by 120 s annealing at 45–65°C and 120 s extension at 72°C. Amplified DNA products were isolated from 1.2% agarose gel and recombined into GATEWAY compatible pDONR221 plasmid (Invitrogen), thus creating the desired entry DNA plasmids. The identity of all PCR products was verified by DNA sequencing. The sequences of the oligonucleotide primers used to generate huntingtin fragments are available at the supplementary Text S1.

Recombination of entry vectors with pACT-DM and pBTM116_D9 plasmids was used to create prey and bait plasmid constructs for Y2H interaction mating, respectively. Recombination of different DNA fragments was checked by BsrGI restriction.

### Y2H Analysis of Huntingtin Fragments

DNA sequences encoding the huntingtin fragments Htt1-506Q23, Htt507-1230, Htt1223-1941, Htt1934-2666, Htt2536-3144 and Htt2721-3144 were sub-cloned into DNA binding domain (baits) and activation domain (preys) Y2H plasmids using GATEWAY technology (Invitrogen) and a matrix of individual MATa and MATalpha yeast strains was generated for systematic interaction mating [68]. Then, yeast strains expressing bait and prey proteins were mixed in 96-well microtiter plates and diploid yeast strains were formed on YPD agar plates. Y2H interactions were scored by the frequency of appearance on the SDIV agar plates and β-galactosidase activity in SDII and SDIV nylon membranes, respectively. Growth in SDII-agar was monitored as a mating control.

### Cell Line, Cell Culture and Western Blot

Human embryonic kidney HEK293 cells were seeded in 96-well plates and cultured in Dulbecco's modified Eagle's medium supplemented with 10% fetal bovine serum at 37°C and 5% CO_2_. Co-transfection of plasmids was done using Lipofectamine 2000 (Invitrogen) following the manufacturer's protocol. The analyses were performed after 48 hours of transfection. For immunoblotting and LUMIER assay, cells were lysed at 4°C for 40 min in 100 µl lysis buffer containing 50 mM HEPES-KOH pH = 7.4, 150 mM NaCl, 0.1% NP40, 1.5 mM MgCl_2_, 1 mM EDTA, 1 mM DTT, 75 Unit/ml Benzonase (Merck) in the presence of protease inhibitor cocktail (Roche Diagnostic). The expression of the constructs was analyzed by Western blot using antibodies against V5-epitope (Invitrogen) and Protein-A (Sigma), while equal protein loading with anti-tubulin antibodies (Figure 3C).

### LUMIER Assay

For LUMIER assay two vectors were generated based on pCDNA3.1(+) (Clontech). For the pPAReni-DM the following cassette was cloned between the BamHI and XbaI sites: Kozak sequence, a double protein A epitope, Renilla Luciferase and the ccdB cassette with flanking R1 and R2 att-sites. For the pFireV5-DM vector the following cassette was cloned between the BamHI and XbaI sites: firefly Luciferase, V5 epitope and the ccdB cassette with flanking R1 and R2 att-sites. (Sequences of cloned inserts are in Supplementary Table S4 in Text S1).

Pairs of PA-Renilla and firefly-V5-tagged huntingtin-fragment fusion proteins were co-expressed in HEK293 cells. Cell extracts were prepared and assessed for the expression of the fusion proteins by immunoblotting and luciferase assays. Protein complexes were isolated from 70 µl cell extracts using 5 µl IgG-coated Dynal magnetic beads (Dynabeads M-280 Sheep anti-Rabbit IgG), subsequently washed with 100 µl PBS, and the binding of the firefly-V5-tagged fusion huntingtin fragment (Co-IP) to the PA-Renilla-tagged fusion huntingtin fragment protein was quantified by measuring the firefly luciferase activity in a luminescence plate reader (TECAN Infinite M200). Renilla activity was also measured as a control for PA-Renilla constructs expression and binding (IP, data not shown). Luciferase activity was measured using the Dual-Glo Luciferase Assay System (Promega) and a luminescence plate reader (TECAN Infinite M200). Each experiment was performed as triplicate transfection.

## Supporting Information

Dataset S1 Annotated sequences used for the training set
(0.14 MB TDS)

Dataset S2 Full length multiple sequence alignment of human huntingtin and representative homologs
(0.19 MB TDS)

Text S1 Supplementary text and supporting figures
(0.63 MB DOC)
