# Supplementary material for: Detection of Alpha-Rod Protein Repeats Using a Neural Network and Application to Huntingtin
Source: PLoS Comput Biol. 2009 Mar 13;5(3):e1000304. doi: 10.1371/journal.pcbi.1000304 (PMC2647740; doi:10.1371/journal.pcbi.1000304)
Supplement: Text S1 — Supplementary text and supporting figures (0.63 MB DOC) [file pcbi.1000304.s003.doc]

**Detection of alpha-rod protein repeats using a neural network and application to huntingtin.**

**Palidwor *et al.***

**Supplementary section.**

**Accessibility of method**

A web server has been created to allow public use of ARD for alpha-rod repeat detection (accessible at http://www.ogic.ca/projects/ard). It processes one protein sequence (or several protein sequences in a FASTA format) and generates a table of the raw output values provided by the neural network per amino acid, as well as a display of the submitted sequences with each amino acid coloured according to its score.

**Details of the neural network algorithm**

The calculation of values at each layer is performed as follows:

$Y_{i} = \sigma(\sum_{j=1}^{N_{i-1}} W_{ij}Y_{j-1}) i=2,3$ where $Y_{i} ,i=1,N_{i}$ are values at the i-th layer, and $W_{ij}$ is the matrix of weight factors. We used N_1_=39, N_2_=3 and N_3_=1.

A sigmoid non-linear function $\sigma(t)=1/(1+\exp(-t))$ is utilized for processing the sums obtained at each layer.

The network was trained with a set of PDB structures (See Table S1). An output of 0.9 was required when the amino acid in the middle of the repeat’s hinge was situated in the middle of the 39 amino acid window, and a value of 0.1 for any other situation. Those values were used rather than one and zero in order to accelerate the convergence of the training process [1]. Backpropagation [2] was employed to train the network.

**Neural Network training and performance.**

A priori we did not know the ideal position or positions to train the network within the hinge of an alpha-rod repeat. In addition, the classification of characteristic positions on the repeats is complicated, especially as they are somewhat heterogeneous in structure and often very difficult to align in sequence. Visual examination of repeats, however, allows identification of the region between the two alpha helices, aided by software which overlays secondary structure information (like CN3D [3] or RasMol [4]). Therefore, we started annotating regions of five residues in those hinges (the complete annotations are available as supplementary Dataset S1). The network (see Methods) was then trained to give an output of 0.90 when the central position of the input window was placed over the five hinge residues and of 0.10 for the remainder. However, when doing this there was no meaningful output variation observed after running the trained network on the training set. Training the network with the central three residues also produced no meaningful output. Only training the network with the central residue of the hinge produced meaningful results: peaks of high score in a background of low scores.

The detected residue was not always the one given in the training set but often one close to it (as in the example of Figure 1). Our review of the hits provided by the network indicated that the position of the selected residue was more accurate than the one provided by visual examination of the structure, which is error-prone. This indicates that the network was really learning. However, we decided not to modify the training set because, as we show below, the results were satisfactory, and to avoid the risk of over-fitting to our small set of training sequences.

The performance of the training procedure was evaluated by monitoring the behavior of the total network error. The decrease of this value was monotonic, and 25-30 training cycles were required to achieve error convergence. In total, the whole training cycle takes 3-5 minutes for a workstation Pentium(R) 4 CPU 2.93Ghz. The trained neural network was used to analyze proteins at an average rate of 1.6 seconds per protein, as implemented on a sun v60x with dual 3Ghz Xeon processors and 2GB RAM. The neural network was implemented in Fortran 90, compiled using gFortran 4.3.0 (http://gcc.gnu.org/fortran/) and executed on a Linux platform. The ARD server at http://www.ogic.ca/projects/ard is written in Perl 5.8.0 using BioPerl libraries (http://www.bioperl.org) running under an Apache 2.0 web server.

As an initial test, we examined the recall of the network in identifying the sequences used in the training set. The network produced a single numerical score per residue that ranged between 0.10 and 0.89. As expected, sharp maxima tend to occur for one residue per repeat, normally the middle one of the five amino acids used in the training set as positives, but not always (see Figure 1).

No detectable scores were obtained for two of the sequences used for the training: 1HU3 and 1IB2. Recall on the whole set was studied to choose a threshold on minimum score and minimum number of hits for automatic selection of good candidates and for evaluation of the method in a large set of sequences with known structures as derived from the PDB database [5]. Selection of sequences with at least three matches of score 0.8 or higher retrieved five out of eight (missing 1HO8, 1DVP, and 1LRV). The five sequences selected are those with HEAT or armadillo repeats suggesting that their structures are more related than the other three that received fewer high-score matches.

**Optimization of the method using proteins of known structure.**

We applied the method to a large set of sequences with annotated secondary structure derived from their known structure (as described in the DSSP database [6]) to gain insight into the precision of the method. The version used (downloaded on December 2007) referred to 42,907 PDB [5] entries with 138,412 sequences (some structures contain multiple proteins). Of them, 13,094 sequences had at least one detectable hit (score above 0.10).

We reviewed the secondary structure of the residues identified to evaluate if the score is associated with secondary structure types expected for the repeats that we are trying to detect (that is, surrounded by alpha helices). For this, we used the DSSP structural codes assigned to residues. The results indicate that the residues with non-trivial scores (score >= 0.15; 19,210) are more often in non alpha or beta conformations (73%) than residues with non significant scores (46%). When hits with increasing scores are studied, alpha and beta conformations become rarer, with bends, turns and disordered structure increasing (Figure S1). Thus, there is a tendency for the scored hits to be in conformations that match that of an alpha-rod repeat hinge.

Regarding the 39 amino acids surrounding the match, we observed a modest increase in the amount of alpha helical conformation, from 35% in the background to 45% around hits with score >= 0.75. These two results suggest that the score has a tendency to be higher in residues with a structural environment consistent with that found in alpha-rod repeat hinges, but this criterion is possibly not sufficient to select true positives. By definition, the type of repeat we are looking for occurs multiple times in the same sequence. Additional criteria that can be used to select true positives are a multiplicity of hits in a sequence and that multiple hits should not occur too close to each other.

In the set of PDB sequences analyzed, 3,723 sequences had one hit above score 0.8, 407 had two, and 128 had three (See Figure S2). Inspection of the matches indicated several false positives with distance between consecutive matches less than 30 amino acids, which is inconsistent with the detection of a 40 amino acids long repeat. Therefore, we took a conservative approach where an additional restriction to count hits as “good” is that they are at least at 30 amino acids from another hit.

1. With this additional condition, a total of 117 sequences were selected if we require three matches, each scored above 0.8, with a minimum spacing of 30 amino acids between hits. Requiring two matches above 0.8 results in the selection of 148 sequences and one match 1,416. Selection by one match was clearly too unrestrictive (as would be expected from the analysis on sequences from the DSSP database described above), and examination of sequences selected with two matches also indicated the presence of many false positives. A threshold of three matches proved to be optimal but generated a number of obvious false positives. Further examination of results suggested many false positives in large proteins, which have an increased likelihood of accumulating scattered spurious hits. Therefore, we added as a further requirement that hits be near other hits, since these repeats tend to occur in tandem arrays without large insertions between them. A distance of 135 (equivalent to 3 to 4 repeats) was found to be optimal.

Using this threshold, 87 sequences from 46 PDB files were selected (Table S2). Many are multiple chains of the same sequence in the same PDB file, or the identical or very similar sequences in different PDB files. Grouping the results by homology yielded 12 groups. Of those, 71 sequences (representing 36 PDB files in 4 groups) were homologous to sequences used in the training set.

The two structures with armadillo repeats used in the positive set had hits but did not fulfill the criteria outlined above. For example, 3BCT has three hits above 0.8 but they are too far apart. For the Armadillo repeat domain of Plakophilin 1 (1XM9) [7], not included in the training set, three hits are recognized but one is far from the other. As we will discuss later, the network is able nevertheless to identify high-score hits in many other armadillo-repeat containing proteins, like Armadillo from *Drosophila melanogaster*, which gives five hits above 0.8 and two more above 0.7, and the human proteins encoded by HSPBP1, JUP, ARMC8, ARMC3 and ARMC4.

Other sequences were selected that are not homologous to those used in the training set. In total these comprise 16 sequences from 10 PDB files, arranged in 8 homology groups. Only one group (or one PDB with two sequences) constituted a false positive (the glutamyl-tRNA(Gln) amidotransferase subunit E from 2D6F) (see details in Table S2). The others are true positives as described in the literature or in databases. We describe them briefly in the next paragraphs.

**yibA** from *Escherichia coli* (1OYZ) is described as a HEAT repeat containing hypothetical protein deposited in the year 2003 but unreported in the literature to date. In 2002 the sequence of a homolog of the yibA protein (SLR1098 from cyanobacteria *Synechocystis sp.,* GenBank id BAA16757) was described to contain a number of HEAT repeats [8]. SLR1098 is homolog both to yibA (BLAST E-value 0.19) and to the HEAT-repeat protein importin beta-1 subunit (BLAST E-value = 0.025).

**Elongation factor eEF3** from *Saccharomyces cerevisiae* (2IWH) (7) is homologous to human GCN1L1 (BLAST E-value = 1e-19), which we already identified as HEAT-repeat containing in 2001 [9].

**Cand1** from *Homo sapiens* (GenBank id 21361794, PDB 1U6G) [10] contains 27 tandem HEAT repeats and its structure has been solved in complex with Cullin and Roc1. Cand1 is similar to HEAT-repeat containing human GCN1L1 (BLAST E-value = 0.12).

**Hspbp1**’s core domain from human Heat shock protein-binding protein 1 (GenBank id 74734730, PDB 1XQR) (11), is similar to armadillo-repeat *Drosophila melanogaster* importin alpha-1 (BLAST E-value = 0.084).

**PH0542A** is a hypothetical protein from *Pyrococcus horikoshii* (GenBank id 14590443, PDB 2DB0) deposited in 2005, which has significant but low sequence similarity to armadillo-repeat importin alpha-2 (BLAST E-value = 0.066).

**Zyg9** from *Caenorhabditis elegans* (2OF3) [11] contains a domain named TOG, which is composed of HEAT repeats and is similar to human CLASP1(13) (BLAST E-value = 8e-4); proteins of this family have similarity to sequences with HEAT repeats, for example to *Drosophila melanogaster* protein phosphatase 2A 65 kDa regulatory subunit (BLAST E-value = 1.3) (discussed in the main manuscript).

**MTH187** from archaea *Methanobacterium thermoautotrophicum* (1TE4) [12] is composed of three alpha-rod repeats, of lengths of approximately 31 amino acids, relatively short in comparison to armadillo repeats or the HEAT repeats in huntingtin [13] (discussed in the main manuscript).

These seven new examples, described in the literature since 2002, have expanded the range of known alpha-rod repeat containing proteins with two non-eukaryotic ones (*E. coli* yibA and *P. horikoshii* PH0542), and added information on how such repeats perform protein-protein interactions (as in the interaction between HEAT-repeat Cand1, with Cullin and Roc1).

1. **Sequences of the oligonucleotide primers used to generate huntingtin fragments**

Htt1-506Q23:

5´-GGGGACAAGTTTGTACAAAAAAGCAGGCTGGATGGCGACCCTGGAAAAGCTGATG-3’

5’-GGGGACCACTTTGTACAAGAAAGCTGGGTGTCAAGTGTGCTGTGACCGTGGCTGTTC- 3’

Htt507-1230:

5´-GGGGACAAGTTTGTACAAAAAAGCAGGCTGGCTGCAGGCGGACTCAGTGGATCTG-3’

5’-GGGGACCACTTTGTACAAGAAAGCTGGGTGTCACCCCAGTGATGAGGATTTACTTGT-3’

Htt1223-1941:

5´-GGGGACAAGTTTGTACAAAAAAGCAGGCTGGACAAGTAAATCCTCATCACTGGGG-3’

5’-GGGGACCACTTTGTACAAGAAAGCTGGGTGTCAGAAGTCCTGTACTGGAGGCTCGTG-3’

Htt1934-2666:

5´-GGGGACAAGTTTGTACAAAAAAGCAGGCTGGCACGAGCCTCCAGTACAGGACTTC -3’

5’-GGGGACCACTTTGTACAAGAAAGCTGGGTGTCAAGCCCGGTGTTTCCTGGAGTTGAC -3’

Htt2721-3144:

5´-GGGGACAAGTTTGTACAAAAAAGCAGGCTGGCTGATGTATGTGACGCTGACAGAA-3’

5’-GGGGACCACTTTGTACAAGAAAGCTGGGTGTCAGCAGGTGGTGACCTTGTGGACATT-3’

**REFERENCES**

1. Brunak S, Engelbrecht J, Knudsen S (1991) Prediction of human mRNA donor and acceptor sites from the DNA sequence. J Mol Biol 220: 49-65.

2. Rumelhart DE, Hinton GE, Williams RJ (1986) Learning representations by back-propagating errors. Nature 323: 533-536.

3. Wang Y, Geer LY, Chappey C, Kans JA, Bryant SH (2000) Cn3D: sequence and structure views for Entrez. Trends Biochem Sci 25: 300-302.

4. Sayle RA, Milner-White EJ (1995) RASMOL: biomolecular graphics for all. Trends Biochem Sci 20: 374.

5. Berman H, Henrick K, Nakamura H, Markley JL (2007) The worldwide Protein Data Bank (wwPDB): ensuring a single, uniform archive of PDB data. Nucleic Acids Res 35: D301-303.

6. Kabsch W, Sander C (1983) Dictionary of protein secondary structure: pattern recognition of hydrogen-bonded and geometrical features. Biopolymers 22: 2577-2637.

7. Choi HJ, Weis WI (2005) Structure of the armadillo repeat domain of plakophilin 1. J Mol Biol 346: 367-376.

8. Morimoto K, Nishio K, Nakai M (2002) Identification of a novel prokaryotic HEAT-repeats-containing protein which interacts with a cyanobacterial IscA homolog. FEBS Lett 519: 123-127.

9. Andrade MA, Petosa C, O'Donoghue SI, Muller CW, Bork P (2001) Comparison of ARM and HEAT protein repeats. J Mol Biol 309: 1-18.

10. Goldenberg SJ, Cascio TC, Shumway SD, Garbutt KC, Liu J, et al. (2004) Structure of the Cand1-Cul1-Roc1 complex reveals regulatory mechanisms for the assembly of the multisubunit cullin-dependent ubiquitin ligases. Cell 119: 517-528.

11. Al-Bassam J, Larsen NA, Hyman AA, Harrison SC (2007) Crystal structure of a TOG domain: conserved features of XMAP215/Dis1-family TOG domains and implications for tubulin binding. Structure 15: 355-362.

12. Julien O, Gignac I, Hutton A, Yee A, Arrowsmith CH, et al. (2006) MTH187 from Methanobacterium thermoautotrophicum has three HEAT-like repeats. J Biomol NMR 35: 149-154.

13. Andrade MA, Bork P (1995) HEAT repeats in the Huntington's disease protein. Nat Genet 11: 115-116.

14. Cuff JA, Barton GJ (2000) Application of multiple sequence alignment profiles to improve protein secondary structure prediction. Proteins 40: 502-511.

15. Huska MR, Buschmann H, Andrade-Navarro MA (2007) BiasViz: visualization of amino acid biased regions in protein alignments. Bioinformatics 23: 3093-3094.

16. Akhmanova A, Hoogenraad CC, Drabek K, Stepanova T, Dortland B, et al. (2001) Clasps are CLIP-115 and -170 associating proteins involved in the regional regulation of microtubule dynamics in motile fibroblasts. Cell 104: 923-935.

1. **Supplementary Figure Captions.**

**Figure S1. Relation between structure and score of hits.** A total of 138,412 sequences considered in the DSSP database (downloaded December 2007) were analyzed of which 13,094 gave at least one hit. Approximately 3,000 residues gave hits with 0.15 <= score < 0.25, 4,500 with 0.25 <= score < 0.75, and 3,000 with score >= 0.75. The secondary structure of the hits according to DSSP indicates that higher scores tend to be located less in regions of organized alpha and beta structure and more in bends, turns and disorganized structure.

**Figure S2. Sequences detected in PDB.** Number of PDB sequences with a given number of hits at or below a specific score threshold. The X-axis is the score threshold, the Y axis (log scaled) is the number of sequences fulfilling the criteria, and the number above each line is the number of hits required per sequence. Note that no hit-spacing constraints have been applied to this analysis.

**Figure S3. Representation of multiple sequence alignments of selected protein families with alpha-rod repeats.** In the six parts of the figure (A-F) the graphs represent an overview of the alignment with red indicating gaps and black or white aligned sequence. In the top view (labeled GPS rich) white indicates regions rich in Glycine, Proline, and Serine residues (small amino acids) where these residues constitute more than 30% in a window of 50 amino acids. In the bottom view (labeled ARD) white indicates approximate positions for hits by ARD (score > 0.10). The lane labeled 2D indicates secondary structure prediction for a representative human sequence indicated by JPRED3 [14] (green for alpha-helix, blue for beta-strand, black for no prediction, red for alignment gaps). Other graphs represent alpha-rod (green ellipses) and other domains. The identifiers of the sequences generally are composed of a database identifier followed by a species name. If fragments are used this is indicated by a number range. The representation of the alignment was generated using an update of the BiasViz software [15]. **(A)** Sequences of the STAG family with human members STAG1, STAG2 and STAG3, also including homologs from mammals to plants and fungi. Secondary structure prediction is mostly alpha-helix. Scattered hits suggest that the full sequence is formed by repeats flanked by disorganized regions. **(B)** Human FRAP1/mTOR and homologs including sequences from mouse, fungi, and plant. Periodic hits and low complexity regions suggest four N-terminal alpha-rods (green shapes). The C-terminal of the family contains other domains. **(C)** Two hypothetical human proteins (LOC165186 and KIAA0423) share an approximate 600 amino acid large C-terminal domain composed entirely of at least 12 alpha-rod repeats preceded by a disorganized region. Whereas LOC165186 seems to be mammalian specific, KIAA0423 (which has an additional N-terminal alpha-rod repeat domain of more than 500 amino acids) has members as distantly related as nematodes and sea anemone (*Nematostella vectensis*). The non-mammalian members of this long version seem to have a much shorter inter-domain linker. **(D)** Human CKAP5 (hCKAP5) and its orthologs in insects to mammals contain five N-terminal alpha-rod domains (numbered here 1-5) with six repeats each, and a C-terminal domain (in yellow), which is alpha-rich but apparently devoid of these repeats. Nematode sequences (homologous to *Caenorhabditis elegans* zyg9) have a duplicated version of domain 2 (2a-2b), domain 5, and the C-terminal domain. The 3D structure of domain 5 in zyg9 was solved and shown to be composed of an alpha-rod of six repeats [11]. **(E)** Sequences of the CLASP family including human CLASP1 (hCLASP1) and CLASP2 (hCLASP2). Secondary structure prediction and repeat prediction suggest that these proteins are composed of four alpha-rods of about six repeats each. The nematode sequences are much shorter in the linkers between domains, and some of them lack the first alpha-rod domain altogether. It was noted that the first of the four domains holds sequence similarity to the TOG domains [16], for example the one in CLASP1 is similar to the third TOG domain in CKAP5 (BLAST E-value 1e-6) or to *Caenorhabditis elegans* Zyg9’s TOG domain (BLAST E-value 8e-4). **(F)** Human huntingtin and homologs. We identified three domains of repeats. The alignment is provided as supplementary Dataset S2. Identifiers and species: human, *Homo sapiens*; Canis, *Canis familiaris*; Mouse, *Mus musculus*; Monodelphis, *Monodelphis domestica* ; Gallus, *Gallus gallus*; Frog, *Xenopus laevis*; Danio, *Danio rerio*; Tetraodon, *Tetraodon nigroviridis*; Fugu, *Takifugu rubripes*; Gasterosteus, *Gasterosteus aculeatus*; Branchiostoma, *Branchiostoma floridae*; Capitella, *Capitella sp.*; Lottia, *Lottia gigantean* (snail); Nematostella, *Nematostella vectensis*; Trichoplax, *Trichoplax adhaerens*; Cioin, *Ciona intestinalis*; Ciosa, *Ciona savignyi*; Honeybee, *Apis mellifera*; Wasp, *Nasonia vitripennis*; Tribolium, *Tribolium castaneum*; Helobdella, *Helobdella robusta*; Dicdi, *Dictyostelium discoideum*; Monosiga, *Monosiga brevicollis*; Culex, *Culex pipiens*; Aedes, *Aedes aegypti*; Anopheles, *Anopheles gambiae*; Fly, *Drosophila melanogaster*; Brugia, *Brugia malayi*; Q623B6_Caeb, *Caenorhabditis briggsae*; Caere, *Caenorhabditis remanei*; Caeel, *Caenorhabditis elegans*; Naegleria, *Naegleria fowleri*.

**Supplementary Tables**

**Table S1. PDB Training set**

| **Positives** | |  |  |
| --- | --- | --- | --- |
| **PDB** | **Sequence^1^** | **Structure^2^** | **description** |
| 2VGL:B | AP2B1_HUMAN | HEAT ADB | AP-2 complex subunit beta-1 |
| 1QBK:B | TNPO1_HUMAN | HEAT IMB | Transportin-1 |
| 1B3U:A | 2AAA_YEAST | HEAT AAA | Protein phosphatase PP2A regulatory subunit A |
| 3BCT | CTNB1_MOUSE | armadillo | Importin subunit beta-1 |
| 1EE4 | IMA1_YEAST | armadillo | Importin subunit alpha |
| 1LRV | 142350 | other | leucin rich variant A. vinelandii |
| 1HO8 | VATH_YEAST | other | Vacuolar ATP synthase subunit H |
| 1DVP:A | 15291889 | other | VHS domain *D. melanogaster* Hrs |
| 1HU3 | 9967557 | other | middle domain of Eif4Gii |
| 1IB2 | 58257646 | other | pumilio homology |
|  |  |  |  |
| **Negatives** | |  |  |
| **PDB** | **Sequence** | **structure** | **description** |
| 1EGD:A | ACADM_HUMAN | four helical up and down bundle | Medium-chain specific acyl-CoA dehydrogenase |
| 1BPY:A | DPOLB_HUMAN | all alpha class SAM domain like | DNA polymerase beta |
| 1CHU | NADB_ECOLI | spectrin repeats | L-aspartate oxidase |
| 1AUA | SEC14_YEAST | all alpha class RuvA C-terminal domain like | SEC14 cytosolic factor |
| 1MHY:B | MEMB_METTR | ferritin like | Methane monooxygenase component A beta chain |
| 1FTS | FTSY_ECOLI | bromodomain like | Cell division protein ftsY |
| 1CPO | PRXC_CALFU | Ef-hand like | Chloroperoxidase |
| 1BMT:A | METH_ECOLI | methionine synthase domain | Methionine synthase |
| 1TL2 | TAL2_TACTR | all beta | Tachylectin-2 |

^1^Swissprot identifiers except numbers (which are GenBank identifiers).

^2^A detailed annotation per residue is given as supplementary Dataset S1.

**Table S2. Results of predictions in PDB**

| **Description^1^** | **PDB^2^** | **repeat** | **Pfam** | | | | **SMART** | | | **ARD** |
| --- | --- | --- | --- | --- | --- | --- | --- | --- | --- | --- |
|  |  |  | PF00514 | PF02184 | PF02984 | PF03130 | SM00185 | SM00567 | SM00386 |  |
|  |  |  | ARM | HAT | HEAT | PBS | ARM | PBS | HAT |  |
| Protein phosphatase (14/7) | **1B3U:A** | HEAT |  |  | 11 |  |  |  |  | 11 |
|  | 1B3U:B | HEAT |  |  |  |  |  |  |  | 11 |
|  | 2IAE:A | HEAT |  |  |  |  |  |  |  | 11 |
|  | 2IAE:D | HEAT |  |  |  |  |  |  |  | 11 |
|  | 2IE3:A | HEAT |  |  |  |  |  |  |  | 11 |
|  | 2IE4:A | HEAT |  |  |  |  |  |  |  | 11 |
|  | 2NPP:A | HEAT |  |  |  |  |  |  |  | 11 |
|  | 2NPP:D | HEAT |  |  |  |  |  |  |  | 11 |
|  | 2NYL:A | HEAT |  |  |  |  |  |  |  | 10 |
|  | 2NYL:D | HEAT |  |  |  |  |  |  |  | 10 |
|  | 2NYM:A | HEAT |  |  |  |  |  |  |  | 10 |
|  | 2NYM:D | HEAT |  |  |  |  |  |  |  | 10 |
|  | 2PKG:A | HEAT |  |  |  |  |  |  |  | 11 |
|  | 2PKG:B | HEAT |  |  |  |  |  |  |  | 11 |
| Importin alpha (24/16) | 1BK5:A | ARM | 8 |  |  |  | 8 |  |  | 6 |
|  | 1BK5:B | ARM | 8 |  |  |  | 8 |  |  | 6 |
|  | 1BK6:A | ARM | 8 |  |  |  | 8 |  |  | 6 |
|  | 1BK6:B | ARM | 8 |  |  |  | 8 |  |  | 6 |
|  | **1EE4:A** | ARM | 8 |  |  |  | 8 |  |  | 6 |
|  | 1EE4:B | ARM | 8 |  |  |  | 8 |  |  | 6 |
|  | 1EE5:A | ARM | 8 |  |  |  | 8 |  |  | 6 |
|  | 1EJL:I | ARM | 8 |  |  |  | 8 |  |  | 4 |
|  | 1EJY:I | ARM | 8 |  |  |  | 8 |  |  | 4 |
|  | 1IAL:A | ARM | 8 |  |  |  | 8 |  |  | 4 |
|  | 1IQ1:C | ARM | 8 |  |  |  | 8 |  |  | 4 |
|  | 1PJM:B | ARM | 8 |  |  |  | 8 |  |  | 4 |
|  | 1PJN:B | ARM | 8 |  |  |  | 8 |  |  | 4 |
|  | 1Q1S:C | ARM | 8 |  |  |  | 8 |  |  | 4 |
|  | 1Q1T:C | ARM | 8 |  |  |  | 8 |  |  | 4 |
|  | 1UN0:A | ARM | 8 |  |  |  | 8 |  |  | 6 |
|  | 1UN0:B | ARM | 8 |  |  |  | 8 |  |  | 6 |
|  | 1WA5:B | ARM | 8 |  |  |  | 8 |  |  | 6 |
|  | 1Y2A:C | ARM | 8 |  |  |  | 8 |  |  | 4 |
|  | 2C1M:A | ARM |  |  |  |  | 8 |  |  | 4 |
|  | 2C1T:A | ARM | 8 |  |  |  | 8 |  |  | 6 |
|  | 2C1T:B | ARM | 8 |  |  |  | 8 |  |  | 6 |
|  | 2JDQ:A | ARM |  |  |  |  | 8 |  |  | 5 |
|  | 2JDQ:B | ARM |  |  |  |  | 8 |  |  | 5 |
| Transportin-1 (24/15) | 1F59:A | HEAT | 1 |  | 1 |  |  |  |  | 4 |
|  | 1F59:B | HEAT | 1 |  | 1 |  |  |  |  | 4 |
|  | 1GCJ:A | HEAT |  |  | 2 |  |  |  |  | 4 |
|  | 1GCJ:B | HEAT |  |  | 2 |  |  |  |  | 4 |
|  | 1IBR:B | HEAT | 1 |  | 1 |  |  |  |  | 4 |
|  | 1IBR:D | HEAT | 1 |  | 1 |  |  |  |  | 3 |
|  | 1M5N:S | HEAT | 1 |  | 1 |  |  |  |  | 4 |
|  | 1O6O:A | HEAT | 1 |  | 1 |  |  |  |  | 4 |
|  | 1O6O:B | HEAT | 1 |  | 1 |  |  |  |  | 4 |
|  | 1O6O:C | HEAT | 1 |  | 1 |  |  |  |  | 4 |
|  | 1O6P:A | HEAT | 1 |  | 1 |  |  |  |  | 4 |
|  | 1O6P:B | HEAT | 1 |  | 1 |  |  |  |  | 4 |
|  | **1QBK:B** | HEAT |  |  | 6 |  |  |  |  | 7 |
|  | 1QGK:A | HEAT | 1 |  | 1 |  |  |  |  | 3 |
|  | 1QGR:A | HEAT | 1 |  | 1 |  |  |  |  | 3 |
|  | 1UKL:A | HEAT |  |  | 2 |  |  |  |  | 3 |
|  | 1UKL:B | HEAT |  |  | 2 |  |  |  |  | 3 |
|  | 2BKU:B | HEAT |  |  | 2 |  |  |  |  | 4 |
|  | 2BKU:D | HEAT |  |  | 2 |  |  |  |  | 4 |
|  | 2BPT:A | HEAT |  |  |  |  |  |  |  | 4 |
|  | 2H4M:A | HEAT |  |  |  |  |  |  |  | 8 |
|  | 2H4M:B | HEAT |  |  |  |  |  |  |  | 8 |
|  | 2OT8:A | HEAT |  |  |  |  |  |  |  | 8 |
|  | 2OT8:B | HEAT |  |  |  |  |  |  |  | 8 |
| AP-1 complex subunit 2 beta-1 (14/2) | **1GW5:A*** | HEAT |  |  |  |  |  |  |  | 6 |
|  | 1GW5:B | HEAT |  |  |  |  |  |  |  | 8 |
|  | 1W63:A | HEAT |  |  |  |  |  |  |  | 6 |
|  | 1W63:B | HEAT |  |  |  |  |  |  |  | 8 |
|  | 1W63:C | HEAT |  |  |  |  |  |  |  | 6 |
|  | 1W63:D | HEAT |  |  |  |  |  |  |  | 8 |
|  | 1W63:E | HEAT |  |  |  |  |  |  |  | 6 |
|  | 1W63:F | HEAT |  |  |  |  |  |  |  | 8 |
|  | 1W63:G | HEAT |  |  |  |  |  |  |  | 6 |
|  | 1W63:H | HEAT |  |  |  |  |  |  |  | 8 |
|  | 1W63:I | HEAT |  |  |  |  |  |  |  | 6 |
|  | 1W63:J | HEAT |  |  |  |  |  |  |  | 8 |
|  | 1W63:K | HEAT |  |  |  |  |  |  |  | 6 |
|  | 1W63:L | HEAT |  |  |  |  |  |  |  | 8 |
| yibA (1/1) | 1OYZ:A | HEAT |  |  |  |  |  |  |  | 3 |
| Cand1 (1/1) | 1U6G:C | HEAT |  |  | 2 |  |  |  |  | 12 |
| Hspbp1 (4/2) | 1XQR:A | ARM |  |  |  |  |  |  |  | 3 |
|  | 1XQR:B | ARM |  |  |  |  |  |  |  | 3 |
|  | 1XQS:A | ARM |  |  |  |  |  |  |  | 3 |
|  | 1XQS:B | ARM |  |  |  |  |  |  |  | 3 |
| glutamyl-tRNA(Gln) amidotransferase subunit E (2/1) | 2D6F:C | none |  |  |  |  |  |  |  | 3 |
|  | 2D6F:D | none |  |  |  |  |  |  |  | 3 |
| PH0542A (2/1) | 2DB0:A | PBS |  |  |  |  |  |  |  | 3 |
|  | 2DB0:B | PBS |  |  |  |  |  |  |  | 3 |
| eEf2 (4/2) | 2IWH:A | HEAT |  |  |  |  |  |  |  | 4 |
|  | 2IWH:B | HEAT |  |  |  |  |  |  |  | 4 |
|  | 2IX3:A | HEAT |  |  |  |  |  |  |  | 4 |
|  | 2IX3:B | HEAT |  |  |  |  |  |  |  | 4 |
| MTH187 (1/1) | 2OF3:A | PBS |  |  |  |  |  |  |  | 3 |
| undetected by ARD (35/25) | 1EFX:C | ARM | 1 |  |  |  |  |  |  |  |
|  | 1G3J:A | ARM | 6 |  |  |  | 12 |  |  |  |
|  | 1G3J:C | ARM | 6 |  |  |  | 12 |  |  |  |
|  | 1I7W:A | ARM | 6 |  |  |  | 12 |  |  |  |
|  | 1I7W:C | ARM | 6 |  |  |  | 12 |  |  |  |
|  | 1I7X:A | ARM | 6 |  |  |  | 12 |  |  |  |
|  | 1I7X:C | ARM | 6 |  |  |  | 12 |  |  |  |
|  | 1JDH:A | ARM | 6 |  |  |  | 12 |  |  |  |
|  | 1JPP:A | ARM | 6 |  |  |  | 12 |  |  |  |
|  | 1JPP:B | ARM | 6 |  |  |  | 12 |  |  |  |
|  | 1JPW:A | ARM | 6 |  |  |  | 12 |  |  |  |
|  | 1JPW:B | ARM | 6 |  |  |  | 12 |  |  |  |
|  | 1JPW:C | ARM | 6 |  |  |  | 12 |  |  |  |
|  | 1LUJ:A | ARM | 6 |  |  |  | 11 |  |  |  |
|  | 1M1E:A | ARM | 6 |  |  |  | 12 |  |  |  |
|  | 1QZ7:A | ARM | 6 |  |  |  | 12 |  |  |  |
|  | 1T08:A | ARM | 6 |  |  |  | 11 |  |  |  |
|  | 1TE4:A | PBS |  |  |  | 3 |  | 3 |  |  |
|  | 1TH1:A | ARM | 6 |  |  |  | 12 |  |  |  |
|  | 1TH1:B | ARM | 6 |  |  |  | 12 |  |  |  |
|  | 1V18:A | ARM | 6 |  |  |  | 12 |  |  |  |
|  | 1XM9:A | ARM | 4 |  |  |  | 7 |  |  |  |
|  | 2BCT:A | ARM | 6 |  |  |  | 11 |  |  |  |
|  | 2FO7:A | HAT |  |  |  |  |  |  | 3 |  |
|  | 2GL7:A | ARM |  |  |  |  | 12 |  |  |  |
|  | 2GL7:D | ARM |  |  |  |  | 12 |  |  |  |
|  | 2HYZ:A | HAT |  |  |  |  |  |  | 3 |  |
|  | 2OND:A | HAT |  |  |  |  |  |  | 5 |  |
|  | 2OND:B | HAT |  |  |  |  |  |  | 5 |  |
|  | 2OOE:A | HAT |  |  |  |  |  |  | 10 |  |
|  | 2UY1:A | HAT |  |  |  |  |  |  | 4 |  |
|  | 2UY1:B | HAT |  |  |  |  |  |  | 4 |  |
|  | 2Z6G:A | ARM |  |  |  |  | 12 |  |  |  |
|  | 2Z6H:A | ARM |  |  |  |  | 12 |  |  |  |
|  | **3BCT:A** | ARM | 6 |  |  |  | 9 |  |  |  |

^1^Within brackets: number of sequences / number of PDB files.

^2^Sequences detected by ARD have been grouped by sequence homology. Bold indicates used in training set.

^*^PDB identifier 1GW5 has been recently superseded by 2VGL.

**Table S3**. High similarity alpha-alpha repeats in Fungi and Prokaryotes.

| Identifier of representative and species | Taxa common to orthologs | Length (aa) | Consensus^1^ | Number of repeats detected | | |
| --- | --- | --- | --- | --- | --- | --- |
|  |  |  |  | ARD | SMART | PFAM |
| N9414_03573  *Nodularia spumigena CCY9414* | *Nostoc sp. PCC 7120*  Cyanobacteria of the family Nostocaceae | 31 | 90% scIKSEAAIPGLIKLLE**c**EDSsVRhSAA.AL | 12 | 11 EZ_HEAT | 12 HEAT_PBS |
| YP_324010 *Anabaena variabilis ATCC 29413* | *Lyngbya sp. PCC 8106* and *Nostoc sp. PCC 7120*  Cyanobacteria | 31 | 90% stIG.pEsAIPuLLELLK**D**SE.NVRSSAA.AL | 12 | 7 EZ_HEAT | 11 HEAT_PBS |
| YP_324969 *Anabaena variabilis ATCC 29413* | *Nostoc sp. PCC 7120* (two) and *Nostoc punctiforme PCC 73102*  Cyanobacteria of the family Nostocaceae | 34 | 90% A+sFKDsspTLshLKp.sh.**D**pp..VRpsAlQEL | 12 | 7 EZ_HEAT | 11 HEAT_PBS |
| FG08957.1 *Gibberella zeae PH-1* | Fungi of class Sordariomycetes | 33 | 90% GpQShLSDThVAALhELhK**D**cDpshR..AAcAI | 14 | 13 EZ_HEAT | 0 |
| XP_001538033 *Ajellomyces capsulatus NAm1* | Fungi | 33 | 80% GpQsSLsEDALQuLVsLLK**s**Kst.VRpSAA.sL | 10 | 6 EZ_HEAT | 6 HEAT_PBS |
| XP_001261463 *Neosartorya fischeri NRRL 181* | Fungi | 33 | 80% stQssWsPpIhpuVhCpLs**s**sst.VRhAAhpAL | 8 | 2 HEAT | 2 HEAT |
| YP_305345 *Methanosarcina barkeri str. Fusaro* | *Methanosarcina acetivorans*, and *Methanosarcina mazei*  Archaea of the family Methanosarcinaceae | 31 | 80% Gphp.sccshtsLlssl..**-**psthVRhtAspuL | 19 | 18 EZ_HEAT | 12 HEAT_PBS + 1 HEAT |
| NP_742379 *Pseudomonas putida KT2440* | Proteobacteria | 31-32 | 80% ...h....sh..hhsshh.s**c**s.h.VRhtAht.L | 9 | 9 EZ_HEAT | 7 HEAT_PBS |
| All repeats^2^ |  |  | 80% Gp.p..stthh.sLhphht**c**pst.lR.tAstAl  2D -------H-HHHHHHHHHH---HHHHHHHHH-- |  |  |  |

Note 1. Consensus predicted using the consensus tool (84). The underscored letter shows the predicted hinge position. Symbols indicate amino acid type: s = small A,C,D,G,N,P,S,T,V; t = turnlike A,C,D,E,G,H,K,N,Q,R,S,T; p = polar C,D,E,H,K,N,Q,R,S,T; u = tiny A,G,S; c = charged D,E,H,K,R; h= hydrophobic A,C,F,G,H,I,K,L,M,R,T,V,W,Y; += positive H,K,R; -= negative D,E; l= aliphatic I,L,V; .= no consensus.

Note 2. Consensus of full alignment of all repeats and secondary structure prediction using JPRED3 (H = helix).

**Supplementary Table S4.**

Sequences of cloned inserts in pPAReni-DM and pFireV5-DM vectors

**pPAReni-DM**

GGATCCGCCGCCACC**ATG**GTGGACAACAAATTCAACAAAGAACAACAAAACGCGTTCTATGAGATCTTACATTTACCTAACTTAAACGAAGAACAACGAAACGCCTTCATCCAAAGTTTAAAAG

ATGACCCAAGCCAAAGCGCTAACCTTTTAGCAGAAGCTAAAAAGCTAAATGATGCTCAGGCGCCGAAAGTAGACAACAAATTCAACAAAGAACAACAAAACGCGTTCTATGAGATCTTACATTT

ACCTAACTTAAACGAAGAACAACGAAACGCCTTCATCCAAAGTTTAAAAGATGACCCAAGCCAAAGCGCTAACCTTTTAGCAGAAGCTAAAAAGCTAAATGGTGCTCAGGCGCCGAAAGTAGAC

GCGAATTCGATGGCTTCCAAGGTGTACGACCCCGAGCAACGCAAACGCATGATCACTGGGCCTCAGTGGTGGGCTCGCTGCAAGCAAATGAACGTGCTGGACTCCTTCATCAACTACTATGATT

CCGAGAAGCACGCCGAGAACGCCGTGATTTTTCTGCATGGTAACGCTGCCTCCAGCTACCTGTGGAGGCACGTCGTGCCTCACATCGAGCCCGTGGCTAGATGCATCATCCCTGATCTGATCGG

AATGGGTAAGTCCGGCAAGAGCGGGAATGGCTCATATCGCCTCCTGGATCACTACAAGTACCTCACCGCTTGGTTCGAGCTGCTGAACCTTCCAAAGAAAATCATCTTTGTGGGCCACGACTGG

GGGGCTTGTCTGGCCTTTCACTACTCCTACGAGCACCAAGACAAGATCAAGGCCATCGTCCATGCTGAGAGTGTCGTGGACGTGATCGAGTCCTGGGACGAGTGGCCTGACATCGAGGAGGATA

TCGCCCTGATCAAGAGCGAAGAGGGCGAGAAAATGGTGCTTGAGAATAACTTCTTCGTCGAGACCATGCTCCCAAGCAAGATCATGCGGAAACTGGAGCCTGAGGAGTTCGCTGCCTACCTGGA

GCCATTCAAGGAGAAGGGCGAGGTTAGACGGCCTACCCTCTCCTGGCCTCGCGAGATCCCTCTCGTTAAGGGAGGCAAGCCCGACGTCGTCCAGATTGTCCGCAACTACAACGCCTACCTTCGG

GCCAGCGACGATCTGCCTAAGATGTTCATCGAGTCCGACCCTGGGTTCTTTTCCAACGCTATTGTCGAGGGAGCTAAGAAGTTCCCTAACACCGAGTTCGTGAAGGTGAAGGGCCTCCACTTCA

GCCAGGAGGACGCTCCAGATGAAATGGGTAAGTACATCAAGAGCTTCGTGGAGCGCGTGCTGAAGAACGAGCAGCTCGAGGATATCACAAGTTTGTACAAAAAAGCAGGC

- *Gene of Interest* – CCAGCTTTCTTGTACAAAGTGGTGATATCGCGGCCGCTCTAGA

**pFireV5-DM**

GGATCCGCCGCCACC**ATG**GAAGACGCCAAAAACATAAAGAAAGGCCCGGCGCCATTCTATCCGCTGGAAGATGGAACCGCTGGAGAGCAACTGCATAAGGCTATGAAGAGATACGCCCTGGTTC

CTGGAACAATTGCTTTTACAGATGCACATATCGAGGTGGACATCACTTACGCTGAGTACTTCGAAATGTCCGTTCGGTTGGCAGAAGCTATGAAACGATATGGGCTGAATACAAATCACAGAAT

CGTCGTATGCAGTGAAAACTCTCTTCAATTCTTTATGCCGGTGTTGGGCGCGTTATTTATCGGAGTTGCAGTTGCGCCCGCGAACGACATTTATAATGAACGTGAATTGCTCAACAGTATGGGC

ATTTCGCAGCCTACCGTGGTGTTCGTTTCCAAAAAGGGGTTGCAAAAAATTTTGAACGTGCAAAAAAAGCTCCCAATCATCCAAAAAATTATTATCATGGATTCTAAAACGGATTACCAGGGAT

TTCAGTCGATGTACACGTTCGTCACATCTCATCTACCTCCCGGTTTTAATGAATACGATTTTGTGCCAGAGTCCTTCGATAGGGACAAGACAATTGCACTGATCATGAACTCCTCTGGATCTAC

TGGTCTGCCTAAAGGTGTCGCTCTGCCTCATAGAACTGCCTGCGTGAGATTCTCGCATGCCAGAGATCCTATTTTTGGCAATCAAATCATTCCGGATACTGCGATTTTAAGTGTTGTTCCATTC

CATCACGGTTTTGGAATGTTTACTACACTCGGATATTTGATATGTGGATTTCGAGTCGTCTTAATGTATAGATTTGAAGAAGAGCTGTTTCTGAGGAGCCTTCAGGATTACAAGATTCAAAGTG

CGCTGCTGGTGCCAACCCTATTCTCCTTCTTCGCCAAAAGCACTCTGATTGACAAATACGATTTATCTAATTTACACGAAATTGCTTCTGGTGGCGCTCCCCTCTCTAAGGAAGTCGGGGAAGC

GGTTGCCAAGAGGTTCCATCTGCCAGGTATCAGGCAAGGATATGGGCTCACTGAGACTACATCAGCTATTCTGATTACACCCGAGGGGGATGATAAACCGGGCGCGGTCGGTAAAGTTGTTCCA

TTTTTTGAAGCGAAGGTTGTGGATCTGGATACCGGGAAAACGCTGGGCGTTAATCAAAGAGGCGAACTGTGTGTGAGAGGTCCTATGATTATGTCCGGTTATGTAAACAATCCGGAAGCGACCA

ACGCCTTGATTGACAAGGATGGATGGCTACATTCTGGAGACATAGCTTACTGGGACGAAGACGAACACTTCTTCATCGTTGACCGCCTGAAGTCTCTGATTAAGTACAAAGGCTATCAGGTGGC

TCCCGCTGAATTGGAATCCATCTTGCTCCAACACCCCAACATCTTCGACGCAGGTGTCGCAGGTCTTCCCGACGATGACGCCGGTGAACTTCCCGCCGCCGTTGTTGTTTTGGAGCACGGAAAG

ACGATGACGGAAAAAGAGATCGTGGATTACGTCGCCAGTCAAGTAACAACCGCGAAAAAGTTGCGCGGAGGAGTTGTGTTTGTGGACGAAGTACCGAAAGGTCTTACCGGAAAACTCGACGCAA

GAAAAATCAGAGAGATCCTCATAAAGGCCAAGAAGGGCGGAAAGATCGCCGTGGACGCGAATTCCGCGGGGCTCGACGGTAAGCCTATCCCTAACCCTCTCCTCGGTCTCGATTCTACGCTCGA

GGATATCACAAGTTTGTACAAAAAAGCAGGC*- Gene of Interest -* CCAGCTTTCTTGTACAAAGTGGTGATATCGCGGCCGCTCTAGA

**Supplementary Files**

**Supplementary file: Dataset S1**

**Annotated sequences used for the training set.**

Description of the columns. (1) Amino acid. (2) Secondary structure, letter codes are G = 3_10 helix, H = Alpha helix, T = H-bonded turn (short segment of helix), E = Extended strand, B = Beta bridge (short segment of strand), S = Bend, 0 = Random Coil. (3) 1 = positions in a turn between two helices of an alpha-rod repeat. (4) 1 = positions in an alpha-rod repeat. (5) PDB code of sequence. (6) Amino acid position in sequence.

**Supplementary file: Dataset S2**

**Full length multiple sequence alignment of human huntingtin and representative homologs.**

FASTA format.

**< 0.15**

**0.15 - 0.24**

**0.25 - 0.75**

**>= 0.75**

Figure S1

alpha

beta

turn

bend

disordered

Score

Seq.

1

2

5

4

3

Figure S2


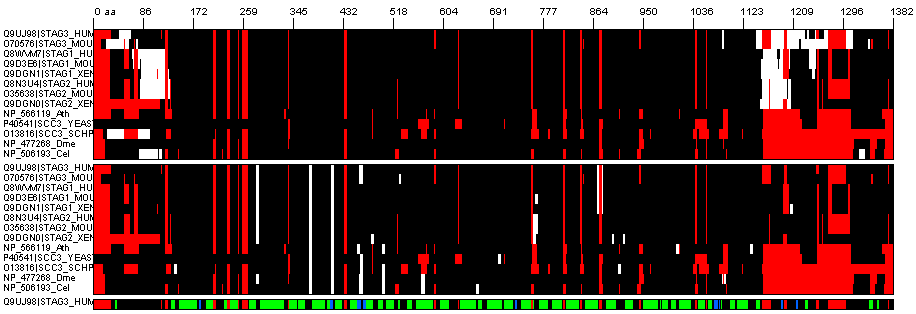


ARD

STAG1/2/3

Figure S3A

2D

GPS-rich


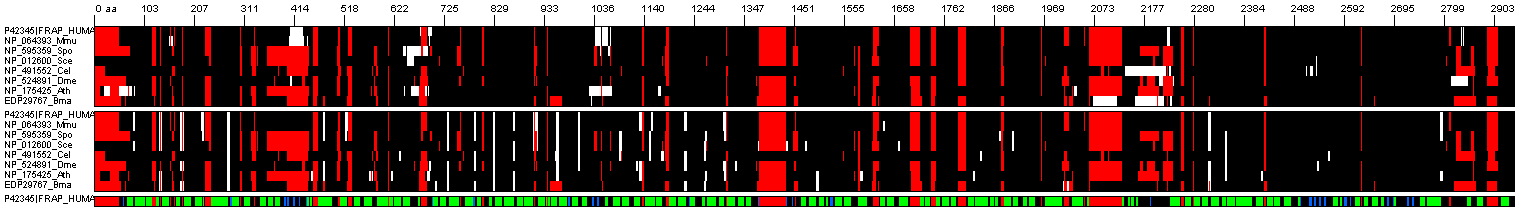


FRAP1

FATc

Rapamycin bind

Figure S3B

GPS

ARD

2D


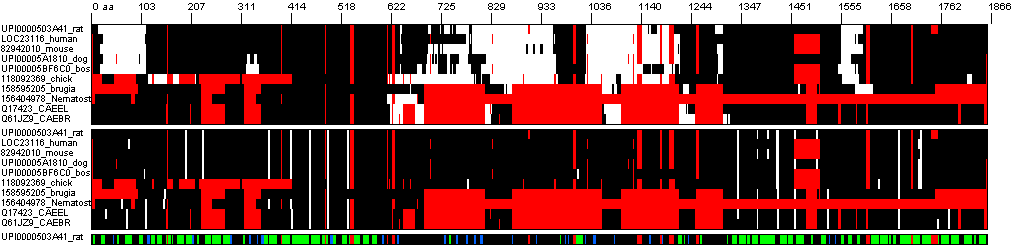


LOC165186

KIAA0423


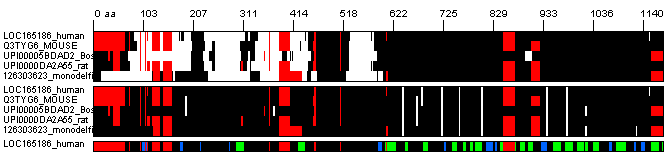


GPS-rich

ARD

GPS-rich

ARD

Figure S3C

2D

2D


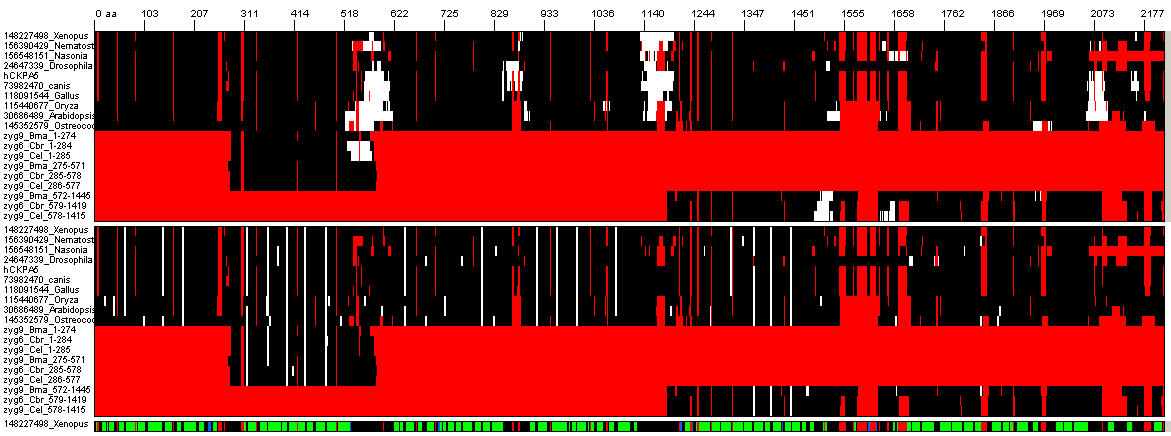


CKAP5 & zyg9

GPS-rich

PDB:2OF3 *C. elegans* zyg9 (602-867)

ARD

Nematode (zyg9)


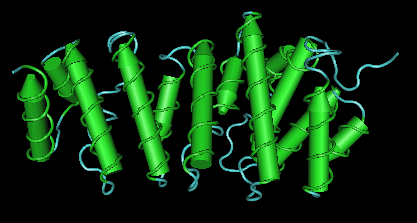


Figure S3D

2D

CLASP1/2

GPS-rich

ARD


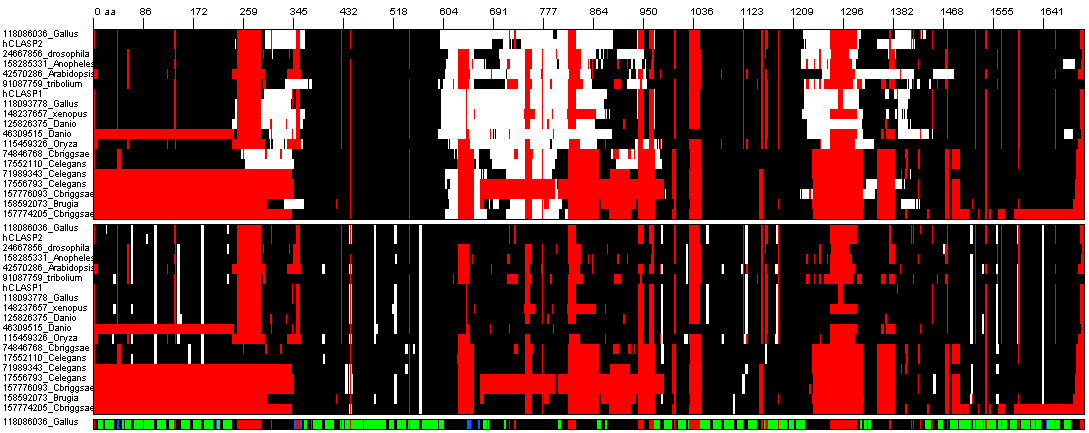


Figure S3E

2D

GPS-rich


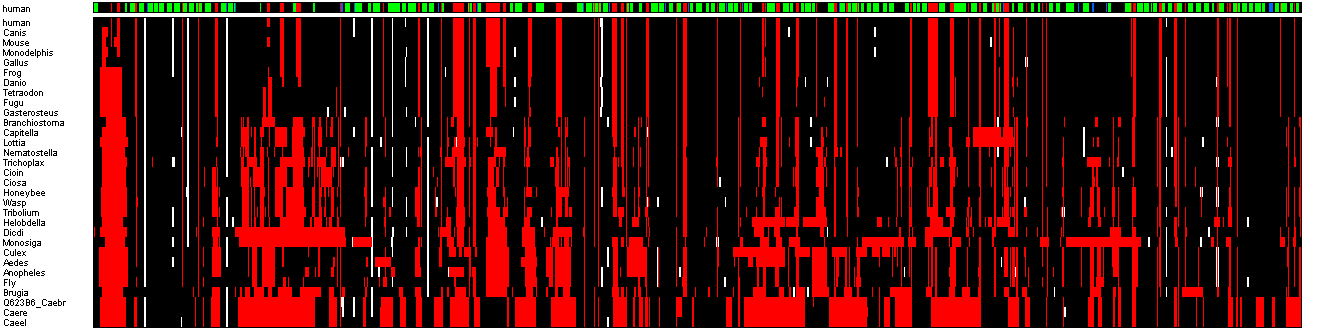

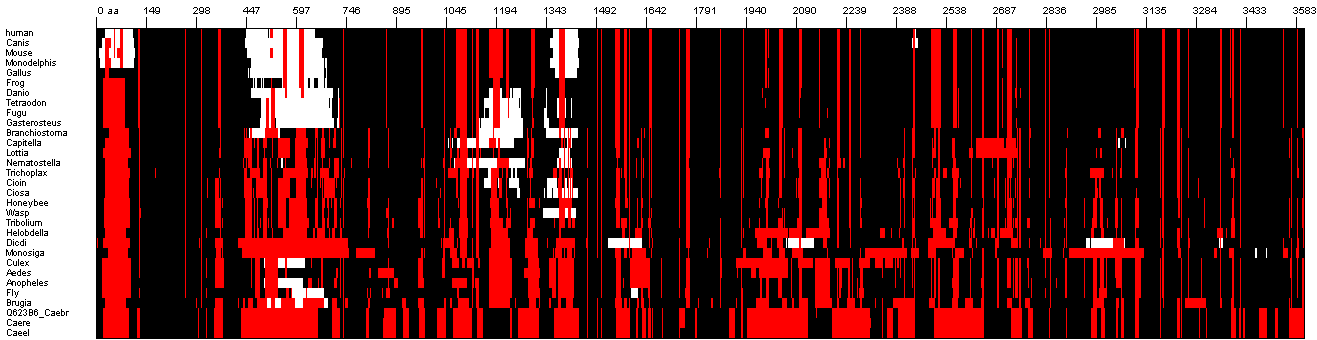


Huntingtin

Figure S3F

ARD

2D

H3

2667-2938

H2

672-969

H1

114-431
